# Purified Sika deer antler protein attenuates GM-induced nephrotoxicity by activating Nrf2 pathway and inhibiting NF-κB pathway

**DOI:** 10.1038/s41598-020-71943-6

**Published:** 2020-09-24

**Authors:** Zhenyi Wang, Lulu Wang, Jing Wang, Jiacheng Luo, Haonan Ruan, Jing Zhang

**Affiliations:** 1 College of Chinese Medicine Materials, Jilin Agricultural University, Xincheng road 2888, Changchun, 130118 China; 2 Changchun SCI-TECH University, Changchun, 130600 China; 3 Jilin Province FAW General Hospital, Changchun, 130000 China

**Keywords:** Cell biology, Drug discovery

## Abstract

Although gentamicin is widely used as an antibiotic in clinical practice, it also has some side-effects, such as acute kidney injury, which is a common condition caused by the abuse of gentamicin. Sika deer antler protein (SDAPR) can antagonize drug-induced AKI. Since SDAPR is recognized as an effective part of velvet antler, its components were further separated. Two components named SDAP1 and SDAP2 were obtained. The protective effects of SDAPR, SDAP1 and SDAP2 on GM-induced cytotoxicity to HEK293 and its potential mechanisms were studied. MTT and xCELLigence Real-Time cell analysis showed that SDAPR, SDAP1 and SDAP2 could protect HEK293 cells from GM toxicity. Similarly, SDAPR, SDAP1 and SDAP2 can reduce ROS level, reduce oxidative stress and improve inflammation Further studies have shown that SDAPR, SDAP1 and SDAP2 upregulate the Nrf2/HO-1 pathway by increasing the expression of Nrf2 and HO-1, and down-regulate the NF-κB pathway by reducing the protein expression of NF-κB. Annexin V/PI flow cytometry and Hoechst 33258 staining showed that SDAPR, SDAP1 and SDAP2 inhibited GM-induced apoptosis in HEK293 cells. Western blot analysis showed SDAPR, SDAP1 and SDAP2 decreased expression level of Bax and Cleaved-caspase-3, and increased the expression level of Bcl-2. In addition, we examined the feasibility of SDAP1 and SDAP1 to avoid kidney injury in a GM mouse model. In conclusion, SDAPR, SDAP1 and SDAP2 can be used to prevent GM-induced HEK293 cytotoxicity, probably because they have strong anti-oxidative stress, anti-inflammatory and anti-apoptotic effects. And SDAP1 and SDAP2 can inhibit GM-induced acute kidney injury in mice.

## Introduction

Drug-induced nephrotoxicity often occurs in long-term drug therapy^1^. The nephrotoxicity is difficult to be predicted in the early stage of disease^2^. At the stage of drug development, the incidence of acute kidney injury caused by drugs in the intensive care unit is close to 30–50%^3^. Gentamicin (GM) is an aminoglycoside drug, used to treat bacterial infections for those caused by Gram-negative organisms^4^. Despite its vast therapeutic benefits as an antibiotic drug, its clinical use is limited majorly because of its nephrotoxicity effect^5^. Because GM can cause nephrotoxicity, it is necessary to reduce the dosage of gentamicin or replace it with other drugs. The recent studies indicated that GM induced nephrotoxicity through multiple mechanisms including generation of oxidative stress and apoptosis^6^. Previous studies have shown that GM induces cytotoxicity in vitro and in vivo, resulting in lipid peroxidation, glutathione reduction and significant increases in activities of superoxide dismutase (SOD), catalase (CAT) and glutathione peroxidase (GPX)^7^.

The master cellular sensor for oxidative stress is the nuclear factor (erythroid-derived 2)-like 2 (Nrf2)^8^. Nrf2 is localized in the cytosol and regulated by its inhibitor Kelch-like ECH-associated protein 1 (Keap1). Nrf2 is transcriptionally inactive until Keap1 is dissociated by electrophiles compounds, ROS, or bioactive phytochemicals found in healthy food. However, various oxidant and toxic insults disturb this sequestration, thereby inducing the nuclear translocation of Nrf2 and its binding to the antioxidant response element (ARE) and expression of antioxidant genes, including NADPH-quinone oxireductase 1 (NQO1) and heme oxygenase 1 (HO-1)^9–11^. Under stressed, HO-1 is expressed to protect cells from ROS generation^12^. The enzyme’s role is to catalyze the conversion of heme to biliverdin, iron, and carbon monoxide, to scavenge free radicals directly^13^. Because of persistent oxidative stress, the cell programs its death by apoptosis to prevent degeneration and proliferation in an uncontrollable way^14^. Activation of Nrf2 can alleviate the toxicity related to reactive oxygen species and is an important molecular target for the prevention of renal injury^15^.

It is recorded in the Chinese pharmacopoeia that Sika deer antler (SDA) has the functions of strengthening kidney yang and enriching blood^16^. It acts as a traditional medicine in treating impotence, slippery essence, infertility due to uterine cold, chills and weakness of bones and muscles. SDA contains a variety of substances, proteins are recognized as effective substances. It is reported that SDA extractive can inhibit cisplatin-induced kidney injury^17^. The results of the previous studies found that Sika deer antler protects (SDAP) against acetaminophen-induced acute kidney injury by activating Nrf2/HO-1 pathway^18^.

Whether SDAP can alleviate GM-induced renal toxicity through the Nrf2 pathway has not been reported. In this study, we investigated the protective effects of SDAP on HEK293 cells in GM-induced oxidative stress, inflammation and apoptosis. We hypothesized that SDAPR, SDAP1 and SDAP2 inhibit GM-induced oxidative stress, inflammatory response and apoptosis by activating the Nrf2/HO-1 pathway. And explored the mechanism of SDAP1 and SDAP2 inhibiting acute kidney injury in GM mice.

## Materials and methods

### Sample extraction and isolation of SDAPR

Extraction and separation of SDAPR based on previous methods^18^. The content of SDAPR was 91.89% by Bradford method. SDAPR (100 mg) was dissolved in 10 mL of distilled water, centrifuged at 8,000 rpm for 10 min at room temperature, and loaded into a column of Sephadex G-100 (Yuan Ye bio, Shanghai, China) for further purification, and eluted with purified water. Flow rate is 0.5 mL/min. According to the absorbance at 280 nm, we collected the eluate from test tube 1–20 as SDAP1 and the eluate from test tube 21–40 as SDAP2. Finally, the homogeneous components SDAP1 and SDAP2 are obtained and lyophilized. The purity of SDAP1 purified by Bradford method was 92.85% and the yield was 12.23%. The purity of SDAP2 was 92.79% and the yield was 11.85%. All experimental methods were implemented in accordance with relevant guidelines and regulations of Jilin Agricultural University.

### Native-PAGE

SDAPR was assessed by non cumulative denaturing polyacrylamide gel electrophoresis (Native-PAGE) using 5% accumulation gel and 10% precipitation gel. Mix the test with 5 × sample buffer (v/v = 4:1). At the same time, three samples were electrophoretically analyzed, including total components and two separated components, as well as 10–200 kDa protein markers. The selected voltages are 120 V and 180 V respectively, which are used for stacking and precipitating gels respectively (Bio-Rad, Hercules, CA, USA). In order to reduce the color of the recoloring and improve the color infectivity, the PAGE gel was swaying for 5 min at a speed of 60 to 70 r/min on the rotating cradle. In this way, the PAGE gel was coloured with Coomassie brilliant blue R-250 heavy colorant (Beyotime, Shanghai, China) at the rate of 60–70 r/min for 30 min, and the deionized water was decolorized in the middle stage. Finally, the filter was carried out to obtain the photo (Perfection V700 Photo, Epson, Suwa, Japan).

### LC–MS/MS and MS data analysis

Following Ruan's method of publishing a paper in 2019^18^, the Native-PAGE gel was cut into thin slices for further identification. The samples were completely decolorized, lyophilized, 40 μL trypsin was added, and treated at 37 °C for 16–18 h. Analysis of samples using liquid chromatography-mass spectrometry/mass spectrometry (Thermo Fisher Scientific, Waltham, MA, USA).

### Database search and bioinformatics analysis

Proteins were identified using Sequest and Proteome Discoverer 1.4 software (Thermo Fisher Scientific, USA). The database used was the uniprot-Cervus nippon transcriptome database. Gene Ontology (https://www.geneontology.org, GO) functional annotation analysis, KEGG signal pathway analysis (https://www.kegg.jp/kegg/pathway.html), and phylogenetic tree analysis of selected differentially expressed proteins.

### Chemicals and regents

GM was purchased from Sigma (purity ≥ 95%; Changchun Amendment Pharmaceutical Co., China). DMEM/HIGH Glucose (DMEM), penicillin/streptomycin and phosphate buffered saline (PBS) were purchased from Hyclone (USA). Then, fetal bovine serum was supplied by Clark Biotechnology institution (USA). 3-(4,5-dimethyl-2-thiazolyl)-2,5-diphenyl-2-H-tetrazolium bromide (MTT), dimethyl sulfoxide (DMSO), Hoechst 33,258 staining solution, Reactive oxygen species fluorescence probe, supplied by Solarbio Life Science Biotechnology (Beijing, China). Superoxide dismutase (SOD), lactate dehydrogenase (LDH), glutathione (GSH), Catalase (CAT) and malondialdehyde (MDA) commercial kits were obtained from Nanjing Jiancheng Biotechnology (Nanjing, China). TNF-α, and IL-6 Elisa kits were purchased from Meilian Biotechnology (Shanghai, China). RIPA lysis buffer and BCA protein assay kits were supplied by Beyotime Biotechnology (Shanghai, China). Primary antibodies of Bax (1:1,000 dilutions), Bcl-2 (1:1,000 dilutions), NQO1 (1:1,000 dilution), Nrf2 (1:1000dilution), HO-1 (1:1,000 dilution), keap1 (1:1,500 dilutions), NF-κB p65 (1:1,000 dilutions),P53 (1:1,000 dilutions), Lamin B (1:1,000 dilutions), caspase-3 (1:1,000 dilutions), Cleaved-Caspase-3 (1:1,000 dilutions) and β-actin (1:1,000 dilutions) were supplied by Solarbio (China).

### In vitro experiments

#### Cell culture and treatment

HEK293 cells were purchased from the American Type Culture Collection (ATCC, Manassas, VA, USA) and conserved at the Laboratory of Molecular Biology in Jilin Agricultural University, maintained in Dulbecco's Modified Eagle Medium (DMEM, Hyclone) supplemented with 10% fetal bovine serum (FBS, Clark, USA) at 37 °C in a 5% CO2 atmosphere. Cell culture was done as portrayed above, and HEK-293 cells in logarithmic development stage were vaccinated into 96-well plates or 6-well plates at a cell number of 5 × 10^4^ cells/mL. Following 24 h of culture, the cell thickness was 80%. Pretreatment of various parts of SDAPR was completed 24 h preceding the use of GM. Control cells were treated with PBS at a demonstrated focus and time.

#### Cell growth assay

Cell viability was determined by a quantitative colorimetric assay with MTT^19^. To screen the pre-protection of SDAP1 and SDAP2, cells were pretreated with SDAP1 and SDAP2 (0.25, 0.5, 0.75, 1, 1.25, 1.5 mg/mL) before administration of GM (3 mg/mL) for 24 h. HEK-293 cells were dispensed in 96-well plates at a density of 8,000 cells/well and cultured at 37 °C for 24 h. After 24 h incubation, cells were treated with various concentrations of SDAP1 and SDAP2 (0.25, 0.5, 0.75, 1, 1.25 and 1 mg/mL) at 24 h, and afterwards exposed to GM (3 mg/mL) for 24 h. Next, after 24 h of GM treatment, the MTT solution was added to each well, and incubated at 37 °C for 4 h. The medium was removed, and 150 μL of dimethylsulfoxide (Solarbio, China) was added to each well. Finally, the optical density was detected with a microplate reader (Nano, Germany) at 490 nm.

#### Determination of cell growth by RTCA method

According to Szyszka, M's experimental method using Real-Time Cell Analyser to verify the proliferation rate of LNCaP cells, we also used Real-Time Cell Analyser to detect the growth of HEK293 cells^20^. The HEK-293 cells in the logarithmic growth phase were digested to prepare a single cell suspension, and the cell concentration was adjusted to 5 × 10^3^ cells/mL. First, 50 μL of the medium was placed in each well on the E-Plate plate and placed on the RTCA Station. The RTCA automatically scanned and began to check the baseline to confirm that the selected wells are in contact. After the baseline of all wells was between 40 and 120, we took out the E-plate and added 100 μL HEK293 cell suspension (5,000 cells/well) into the wells. Incubated for 24 h at 37 °C in a 5% CO_2_ incubator. Subsequently, the treatment group was administered with SDAPR (4 mg/mL), SDAP1 (1 mg/mL) and SDAP2 (1 mg/mL). The GM model group was administered the same amount of PBS. After 18 h, the treatment group and the model group were given GM (3 mg/mL) simultaneously, the control group was given the same amount of PBS. We put the E-Plate plate on the RTCA Station in the cell incubator, and the system detected the Cell Index value of the cells.

#### LDH leakage assay

To evaluate plasma membrane integrity, LDH release was quantified using a commercial colorimetric assay kit (Nanjing Jiancheng Biotechnology Institute, Nanjing, China) according to the manufacturer's instructions.

#### Detection of total GSH

To detect GSH content in HEK293 cells, HEK-293 cells (2 × 10^5^ cells/pore) were inoculated into 6-well plate. After 24 h of incubation, HEK-293 cells were pre-incubated for 12 h in the presence of SDAPR (1, 2, 4 mg/mL), SDAP 1 and SDAP2 (0.25, 0.51 mg/mL), and then exposed to GM (3 mg/mL) for 24 h. The determination was carried out according to the reduced glutathione (GSH) assay kit from Solarbio (Beijing, China).

#### Lipid peroxidation

MDA levels were measured using assay kits from Nanjing Jiancheng Bioengineering Institute (Nanjing, China) according to the manufacturer's protocols. Briefly, HEK-293 cells (2 × 10^5^ cells/well) were plated into 6-well plates. After incubation for 24 h, HEK-293 cells were pre-incubated in the presence of SDAPR (1, 2, 4 mg/mL), SDAP1 and SDAP2 (0.25, 0.5 1 mg/mL) for 12 h, and then the cells were exposed to GM (3 mg/mL) for 24 h. Cells were collected and MDA levels in HEK293 cells were measured according to manufacturer's instructions.

#### Measurement of superoxide dismutase activity

In order to determine the activity of SOD in HEK293 cells, HEK-293 cells (2 × 10^5^ cells/pore) were inoculated into 6-well plate. After 24 h of incubation, HEK-293 cells were pre-incubated for 12 h in the presence of SDAPR (1, 2, 4 mg/mL), SDAP 1 and SDAP2 (0.25, 0.51 mg/mL), and then exposed to GM (3 mg/mL) for 24 h. The determination was carried out according to the SOD kit instructions from Nanjing Jiancheng Bioengineering Institute (Nanjing, China).

#### Measurement of catalase activity

In order to determine the activity of SOD in HEK293 cells, HEK-293 cells (2 × 10^5^ cells/pore) were inoculated into 6-well plate. After 24 h of incubation, HEK-293 cells were pre-incubated for 12 h in the presence of SDAPR (1, 2, 4 mg/mL), SDAP 1 and SDAP2 (0.25, 0.51 mg/mL), and then exposed to GM (3 mg/mL) for 24 h. The determination was carried out according to the SOD kit instructions from Solarbio Bioengineering Institute (Beijing, China).

#### Determination of intracellular reactive oxygen species (ROS) level

DCFH-DA method was used to detect active oxygen content. Cells were cultured in 6-well plates at 8,000 per well. Subsequently, it was stained with 20 μm DCFH-DA (Solarbio, Beijing, China) in the dark for 1 h, and then the fluorescence intensity was detected by fluorescence spectrophotometry. The excitation and emission wavelengths were 485 and 530 nm, respectively.

#### Mitochondrial membrane potential (ΔΨm) assay

Alteration of HEK293 cells in the mitochondrial membrane potential were assayed using a mitochondrial membrane potential assay kit with JC-10 (Solarbio Life Science, China) according to manufacturer’s instructions. In short, cells were pretreated with various concentrations of SDAPR in 6-well pate at a denseness of 2 × 10^5^ cells/well for 24 h, and then exposed to GM (3 mg/mL) for 24 h. Cells were fostered within JC-10 staining working solution (1 mL) for 20 min at 37 °C in dark. The result was emerged by a fluorescence microscope and analyzed by fluorescence microplate reader.

#### ELISA

Determination of TNF-α and IL-6 in GM-treated HEK293 cells by enzyme-linked immunosorbent assay (mlbio, Shanghai, China).

#### Hoechst 33258 staining assay

To determine whether SDAPR could increase viability of HEK293 after GM treatment is mainly based on inhibition of apoptotic on nuclear morphology changes which were determined by using Hoechst 33258 staining. According to the commercial kit instruction (Solarbio Life Science, China) and measured with a fluorescence microscope (Leica DMI3000 B, Germany). Cells with the characteristic nuclear changes of chromatin condensation and nuclear fragmentation were considered apoptotic.

#### Protein extraction and western blotting

We collected the treated HEK293 cells, washed them once with PBS, added the proteolytic lysate, and lysed the cells on ice. The cells were centrifuged at 4 ℃ and 13,000 r/min for 10 min. Store in tube at − 80 °C. The BCA protein concentration test was used to calculate the protein concentration of the sample. Separated on a 10% SDS polyacrylamide gel. It is then electrically transmitted to the PVDF membrane. Before incubating the primary antibody, tailor the PVDF membrane according to the molecular weight range of the target antibody. Use anti-HO-1 (wanleibio, China), anti-Nrf2 polyclonal (wanleibio, China), anti-NF-κB, anti-NQO1 (Solarbio, China), anti-keap1 (wanleibio, China), anti-P53 (wanleibio, China), anti-Lamin B, anti-caspase-3 (Solarbio, China) and cleaved caspase-3 (Solarbio, China) for western blotting. Finally, the cut PVDF membrane was placed on a plastic wrap and an appropriate amount of ECL kit was taken. Medium volumes of liquids A and B were mixed, mixed and added to the surface of the membrane, transferred to a gel imaging analyzer, and exposed in a chemical light-sensitive mode. After exporting photos in TIF format, analyze the optical density of each band under ImageJ software^18^.

#### Measurement of cell viability by flow cytometry using annexin V/PI staining

The HEK-293 cells were dispensed onto a six-well microplate at a concentration of 5 × 10^4^ cell/well. After 24 h of incubation, HEK-293 cells were pre-incubated for 12 h in the presence of SDAPR (1, 2, 4 mg/mL), SDAP1 and SDAP2 (0.25, 0.51 mg/mL), and then exposed to GM (3 mg/mL) for 24 h. The cells were analyzed using a flow cytometer.

### In vivo experiments

#### Animals and experimental protocol

Healthy SPF C57BL/6 mice, male, 8 weeks old, weighing 18–22 g, animal production license number: SCXY-2011-0004), were purchased from Changchun Hongda Experimental Animal Co., Ltd. The mice were housed in a laminar flow rack, freely eating and drinking water, maintaining constant temperature conditions, and a relative humidity between 50 and 60%. Thirty mice were randomly divided into 6 groups by a random number table method, with 5 mice in each group. All animal experiments were approved by the Animal Ethics Committee of Jilin Agricultural University.

Group I was used as a control group, and the same amount of distilled water (SDAP vector; p.o.) was administered for 10 consecutive days. After the oral administration of distilled water on the tenth day, a similar amount of normal saline (GM vector; i.p.) was administered and 5 consecutive days.

Group II was fed with an equivalent amount of distilled water for 10 consecutive days (SDAP vector; p.o.). From the tenth day, a similar amount of GM (100 mg/kg; i.p.) dissolved in normal saline was administered for 5 consecutive days.

Groups III and IV were given SDAP1 at low dose (15 mg/kg; p.o.) or high dose (60 mg/kg; p.o.) for 10 consecutive days, respectively. From the tenth day, SDAP1 was administered orally and then a similar amount of GM (100 mg/kg; i.p.) dissolved in physiological saline was administered for 5 consecutive days.

Group V and VI were administered low dose (15 mg/kg; p.o.) or high dose (60 mg/kg; p.o.) SDAP2 for 10 consecutive days, respectively. From the tenth day, SDAP1 was administered orally and then a similar amount of GM (100 mg/kg; i.p.) dissolved in physiological saline was administered for 5 consecutive days.

During the whole period of administration, the daily activities of diet and drinking water and changes in body weight of the mice were recorded, and the urine of each group of mice was collected by the metabolic cage method and stored for future use. All animals were fasted for 12 h before administration on day 15, and 16 h after administration, blood was collected from the orbital iliac veins and sacrificed. Renal tissues were taken in an ice bath, kidney appearance pictures were taken, and kidney weights were recorded. The left kidney was used for the determination of biochemical indicators, and 10% formalin in the right renal vesicle was embedded in paraffin for HE pathological section.

#### Estimation of body and renal weight in mice

During the experiment, the weight, posture and activity of each group of mice were recorded every day. After 10 days of continuous administration, the weight of the mice was weighed, the mice were killed, the kidneys were quickly removed, and the residual blood was dried with filter paper. The kidney index of mice was calculated by weighing the mass, and the kidney coefficient = (kidney mass/body weight) × 100%.

#### Determination of kidney injury biomarker

The fresh blood was placed at room temperature, coagulated and centrifuged at 3,000 r/min for 15 min to separate the serum. The kit was used to detect the contents of Cr and BUN (Jian cheng, Nanjing, China). Determine the relevant OD value according to the instructions of the kit, and then calculate the content.

#### HE staining

The kidney tissue was fixed in a 10% formalin solution and embedded in paraffin, and then the fixed tissue was cut to a thickness of 4 to 5 μm. According to standard methods, hematoxylin–eosin (HE) staining, neutral gum seals, and observation under a 200 × microscope.

#### Western blot analysis

The kidney tissues of each group were frozen with liquid nitrogen and ground into powder. 200 μL of RIPA cell lysate mixed with PMSF and All-in-One was added, lysed on ice for 20 min, and then the protein was collected into a 1.5 mL EP tube. After centrifugation at 8,000 rpm for 15 min at 4 °C, the supernatant was taken, and the protein concentration in the BCA protein quantitative assay kit was adjusted and stored at − 80 °C until use. We followed the steps of 2.6.12 to detect the expression of related proteins.

### Statistical analysis

The results are presented as means ± SEM. The analyses were performed using SPSS 18.0 and the differences were compared with one-way analysis followed by Dunnett’s multiple comparison test *P* < 0.05 was regarded as statistically significant.

### Statement

All experimental methods were implemented in accordance with relevant guidelines and regulations of Jilin Agricultural University.

## Results

### Isolation of SDAPR and identification of SDAP1 and SDAP2 and targets analysis

**Figure 1:**
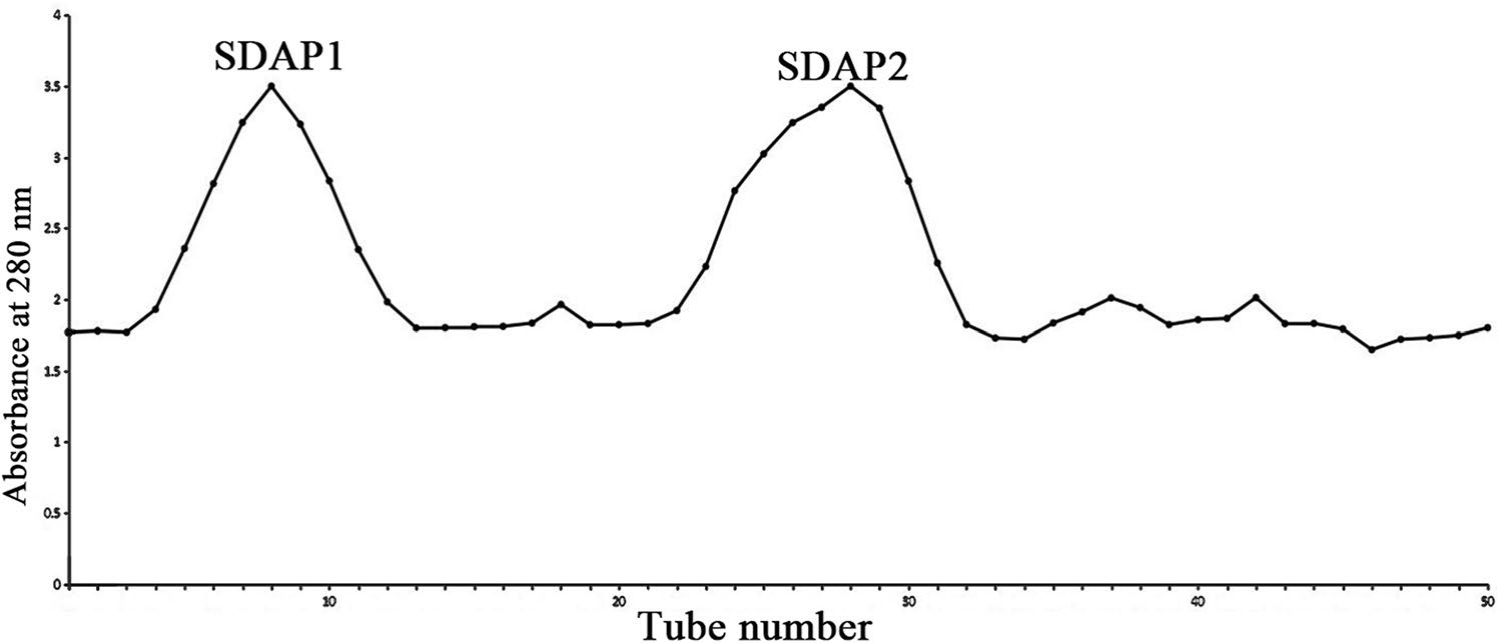
Isolation of SDAPR: elution profile of SDAPR by Sephadex G-100 column.

**Figure 2:**
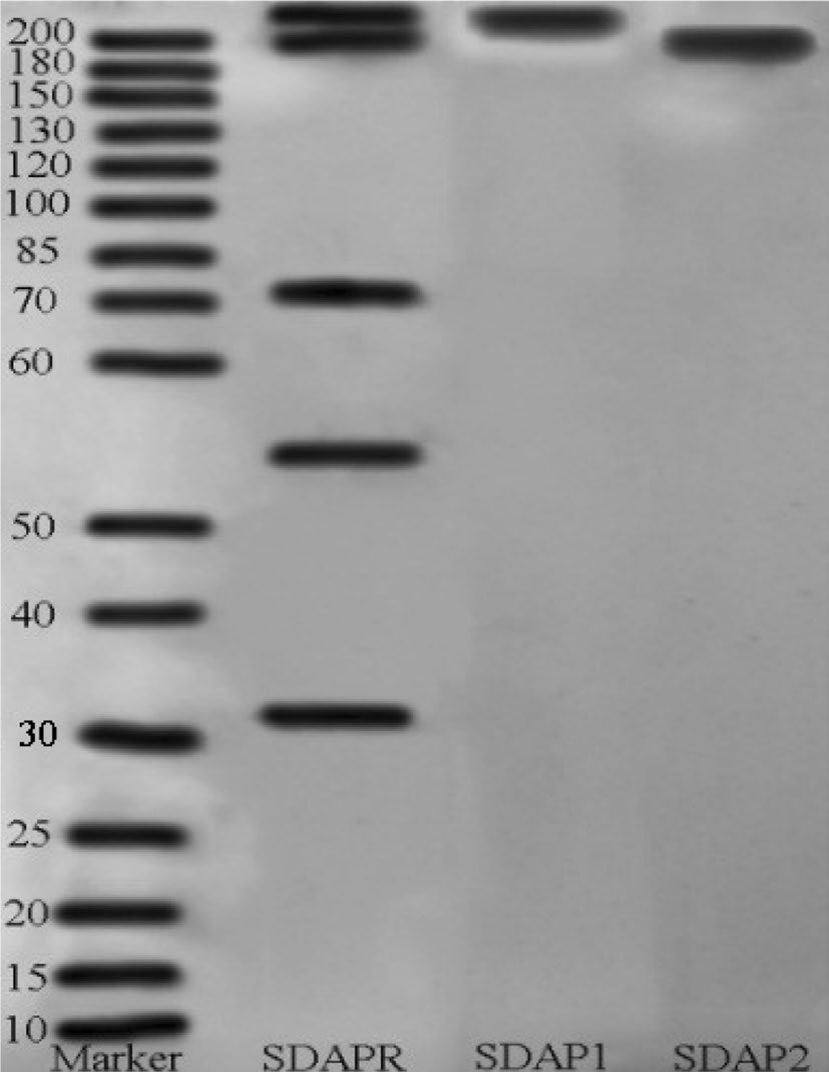
Native-PAGE of different components of SDAPR, SDAP1 and SDAP2.

**Figure 3:**
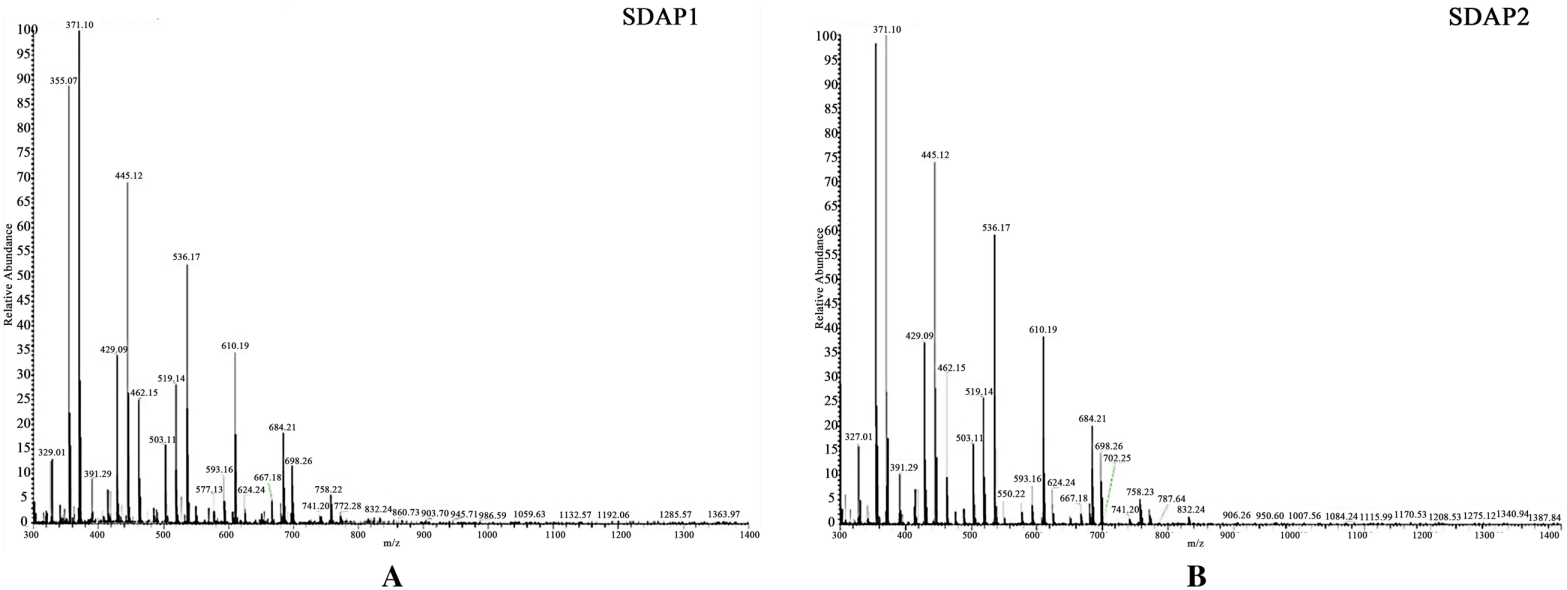
LC–MS/MS images of SDAP1 and SDAP2. (**A**) SDAP1. (**B**) SDAP2.

**Figure 4:**
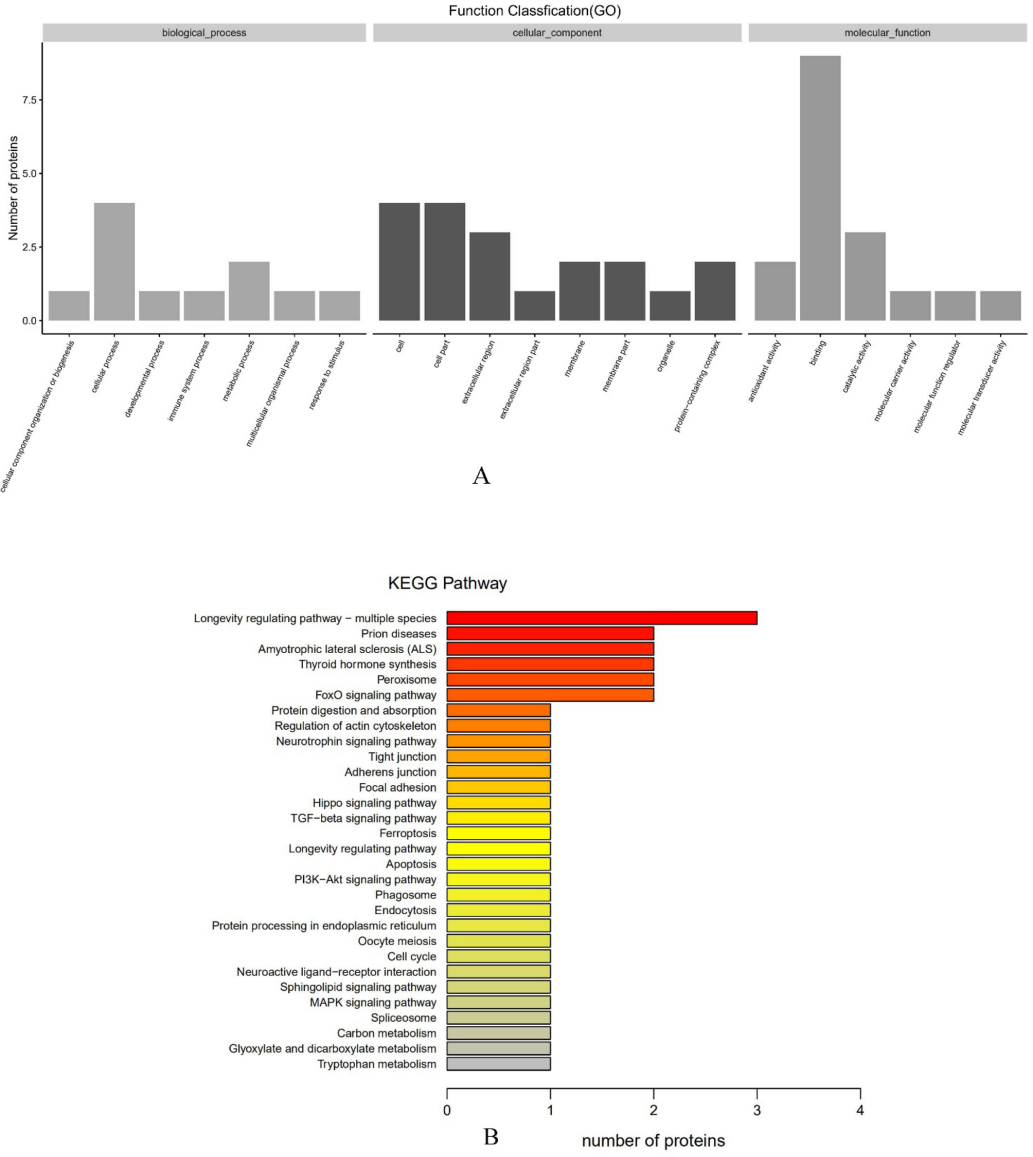
GO and KEGG analysis of SDAPR. (**A**) GO analysis of SDAPR: The horizontal axis provides description of the matched GO terms, whereas the vertical axis is the ratio of proteins in the total identified proteins. (**B**) KEGG analysis of SDAPR: KEGG pathway annotation of tissue-enriched proteins. The horizontal axis is the number of proteins, whereas the vertical ordinates are the terms of the KEGG pathways. KEGG reference source is: https://www.kegg.jp/kegg/kegg1.html.

**Figure 5:**
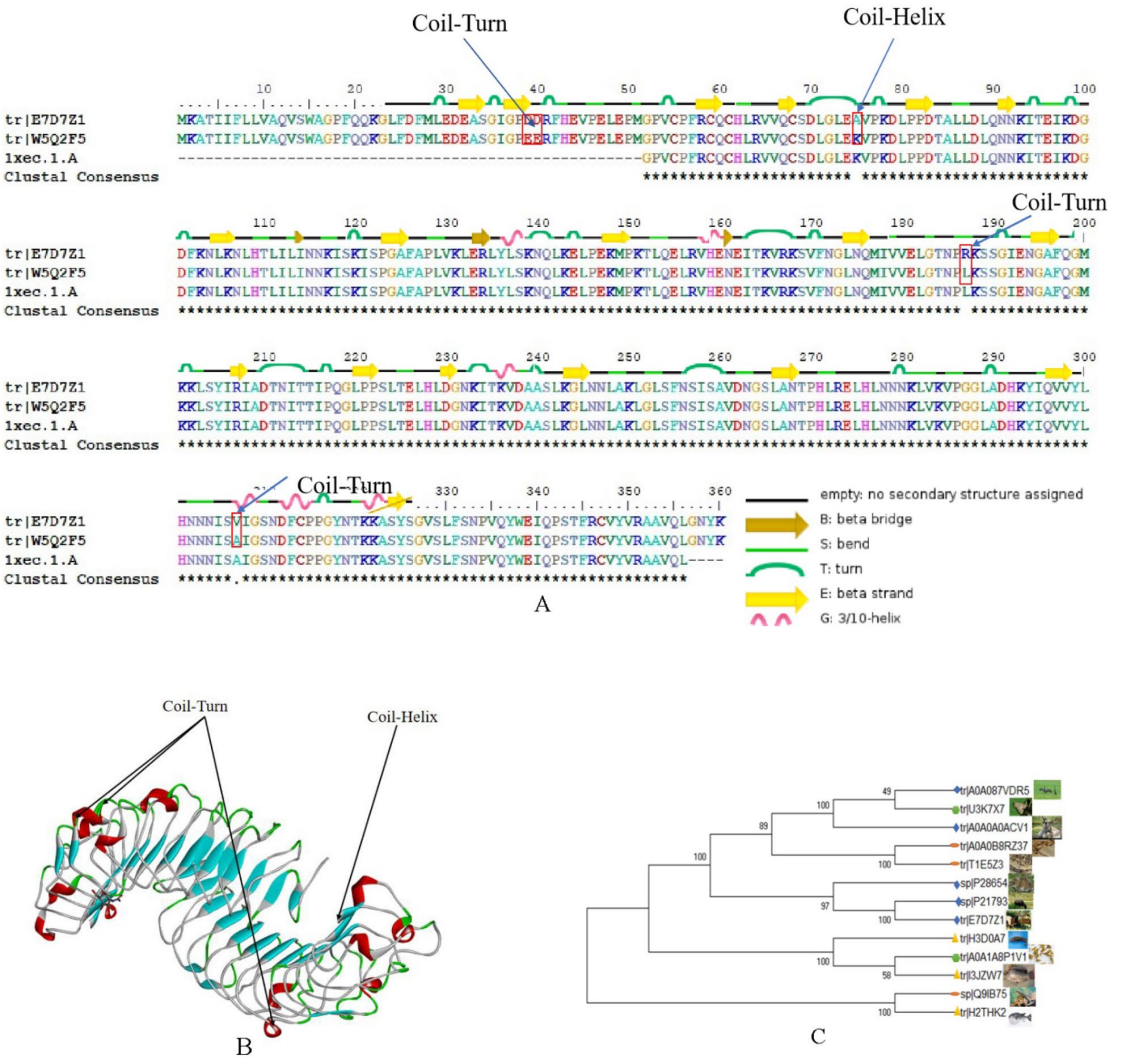
Protein Sequence alignment, 3D modeling and phylogenetic analysis of Decorin from SDAPR. (**A**) A presumed sequence W5Q2F5 aligned with the model Decorin. At the bottom of columns, asterisks (*) show conserved positions, colons (:) show conserved substitutions and points (.) show non-conserved substitutions. Grey line, green bend, blue banded arrowhead and red solenoid represent coil, turn, sheet and helix, respectively. Different fragments are framed by red lines. (**B**) 3D modeling was simulated using the template Decorin by SWISS-MODEL and viewed by Discovery Studio 4.5. The colors grey, green, blue and red represent coils, turns, sheets and helices, respectively. Different fragments are indicated by red arrows. (**C**) Phylogenetic tree constructed using putative Decorin and 13 other sequences from different species using MEGA 7 with the Neighbor-Joining method.

SDAPR was separated by Sephadex G100 column chromatography to obtain SDAP1 and SDAP2 (Fig. 1). SDAPR molecular weight range between 30 and 70 kDa and greater than 200 kDa. The molecular weight range of SDAPR, SDAP1 and SDAP2 is shown in Fig. 2. The mass spectra of SDAP1 and SDAP2 are shown in Fig. 3. Based on the proteomics crude MS information, peptide sequence data was obtained and compared with the proteins in the Uniprot-Cervus nippon database. Using LC–MS/MS technology, 16 trusted proteins were selected from SDAP1, and 11 trusted proteins were selected from SDAP2. The protein identification results of SDAP1 (Table 1) and SDAP2 (Table 2) indicated that the proteins identified in each segment of SDAPR have been effectively separated and independent of the proteins in other parts. Through GO enrichment analysis, the differentially expressed proteins screened were analyzed for biological process, cellular component, and molecular function to study the functional distribution of differentially expressed proteins. The GO analysis (Fig. 4A) showed that 11 differentially expressed proteins were related to biological processes, 19 differentially expressed proteins were related to cellular components, and 17 were related to molecular functions in SDAPR. Here, we found that the protein coverage of the three components related to the molecular functional components is significantly higher than other proteins, including the binding component proteins in SDAP1 and SDAP2, the antioxidant component proteins, and the catalytic active component proteins (Table 3). KEGG analysis showed that the PI3K-AKT pathway is closely related to SDAPR (Fig. 4B). Ongoing examinations have demonstrated that PI3K-Akt pathway assumes a significant job during the time spent intense renal injury. As the downstream pathway of Nrf2 pathway, we speculate that the defense effect of SPAPR on severe kidney damage caused by GM can be determined by Nrf2 guidelines. Combining the results of GO and KEGG analysis, we selected the following an amino acid sequence alignment, three-dimensional structure construction and evolutionary tree mapping of proteins that may protect GM-induced AKI from SDAPR. The accessions of the protein is E7D7Z1 (Fig. 5). After screening different components of the protein, the strip identification and KEGG analysis were carried out. It was found that the structure of E7D7Z1 protein was related to cellular component, molecular function and TGF-beta signaling pathway, and it was found that both E7D7Z1 protein was contained in both SDAP1 and SDAP2. Therefore, proteomic analysis was performed on E7D7Z1 (Table 4). We selected sheep's decoration protein (W5Q2F5) as the control protein, and selected the deer decoration protein (E7D7Z1) as the model arrangement (Fig. 5A) and the basic layout (Fig. 5B). Phylogenetic analysis showed that E7D7Z1 was close to P28654 in Mus musculus (Fig. 5C). Use SWISS-MODEL's Decorin template to simulate 3D modeling and view it with Discovery Studio 4.5 (https://swissmodel.expasy.org/interactive).

**Table 1:** Protein identification results of SDAP1.

Accession	Description	Coverage (%)	Peptides	PSMs	AAs	calc.pI
X2GM95	Serum albumin	66	43	165	583	5.67
A0A2S1M4Y6	Serum albumin	55	32	108	607	6.54
A0A220IG97	Adult beta-globin	78	10	41	145	7.02
J9UJQ1	Annexin	28	7	9	339	7.31
V5LTF3	Cu/Zn superoxide dismutase	19	2	3	151	6.61
G0Z3A2	Catalase	9	3	4	527	7.17
A0A067XP64	Tubulin beta	8	2	2	356	5.86
A4GIN2	Collagen alpha-1 (X)	4	2	2	674	9.6
I0FX03	RNA polymerase beta subunit	12	2	2	275	5.87
A0A2R4PCY0	14-3-3 protein epsilon	4	1	2	255	4.74
A0A0H5BIT5	Heat shock protein 70	3	1	1	635	6.68
F1APU0	Toll-like receptor 3 short-type	4	1	1	250	7.47
A0A0H5BIT0	Actin	6	2	2	334	6.02
E7D7Z1	Decorin	4	1	1	360	8.66
G8FVL2	60 kDa chaperonin	4	1	1	550	5.38
G3M6Y8	Brain-derived neurotrophic factor	8	1	1	189	6.34

*Accession* The accession number of the protein, the accession number of the protein in the proteome in the database that matches the database. The main sources of the database are UniProt/NCBI, databases unique to other species, and customer-supplied databases (derived from transcriptome data). *Description* functional description information of the protein in the database, including the protein name, species name (OS = species Latin name), gene name (GN = **). *Coverage* coverage (percentage), the ratio of the number of amino acids identified by the protein during mass spectrometry to the highest number of amino acids in the proteome. *Peptides* number of peptides, identifying the total number of peptides in a proteome, equivalent to the number of peptides used for qualitative. *PSMs* the number of secondary peptide fragments, which can be used to qualitatively identify the secondary peptide spectrum of the polypeptide. *AAs* number of amino acids, length of protein sequence. *calc.pI* the isoelectric point of the protein.

**Table 2:** Protein identification results of SDAP2.

Accession	Description	Coverage (%)	Peptides	PSMs	AAs	calc.pI	
X2GM95	Serum albumin	67	44	212	583		5.67
A0A2S1M4Y6	Serum albumin	55	35	147	607		6.54
A0A220IG97	Adult beta-globin	69	9	27	145		7.02
J9UJQ1	Annexin	19	5	6	339		7.31
V5LTF3	Cu/Zn superoxide dismutase	19	2	3	151		6.61
A4GIN2	Collagen alpha-1 (X)	2	1	2	674		9.6
I0FX03	RNA polymerase beta subunit	4	1	1	275		5.87
G0Z3A2	Catalase	2	1	1	527		7.17
A0A2R4PCY0	14-3-3 protein epsilon	4	1	1	255		4.74
E7D7Z1	Decorin	3	1	1	360		8.66
A1DRF2	Actin	3	1	1	367		5.33

*Accession* The accession number of the protein, the accession number of the protein in the proteome in the database that matches the database. The main sources of the database are UniProt/NCBI, databases unique to other species, and customer-supplied databases (derived from transcriptome data). *Description* functional description information of the protein in the database, including the protein name, species name (OS = species Latin name), gene name (GN = **). *Coverage* coverage (percentage), the ratio of the number of amino acids identified by the protein during mass spectrometry to the highest number of amino acids in the proteome. *Peptides* number of peptides, identifying the total number of peptides in a proteome, equivalent to the number of peptides used for qualitative. *PSMs* the number of secondary peptide fragments, which can be used to qualitatively identify the secondary peptide spectrum of the polypeptide. *AAs* number of amino acids, length of protein sequence. *calc.pI* the isoelectric point of the protein.

**Table 3:** GO analysis results of SDAP1 and SDAP2.

GO-ID	Class	Term	Protein
GO:0003824	Molecular-function	Catalytic activity	V5LTF3; G0Z3A2
GO:0016209	Molecular-function	Antioxidant activity	V5LTF3; G0Z3A2
GO:0140104	Molecular-function	Molecular carrier activity	A0A220IG97
GO:0032991	Cellular-component	Protein-containing complex	A0A220IG97; A4GIN2
GO:0008152	Biological-process	Metabolic process	G0Z3A2
GO:0044421	Cellular-component	Extracellular region part	X2GM95
GO:0050896	Biological-process	Response to stimulus	G0Z3A2
GO:0005576	Cellular-component	Extracellular region	X2GM95; A4GIN2; E7D7Z1
GO:0005623	Cellular-component	Cell	A0A220IG97; G8FVL2
GO:0005488	Molecular-function	Binding	A0A22II0IG97; V5LTF3; J9UJQ1; G0Z3A2; E7D7Z1; A0A0H5BIT5; G8FVL2
GO:0044464	Cellular-component	Cell part	A0A220IG97; G8FVL2
GO:0098772	Molecular-function	Molecular function regulator	J9UJQ1
GO:0009987	Biological-process	Cellular process	G0Z3A2; G8FVL2

**Table 4:** KEGG analysis results of SDAP1 and SDAP2.

Ko-ID	Ko-description	Pathway-info
ko00380	Tryptophan metabolism	G0Z3A2 (K03781)
ko00630	Glyoxylate and dicarboxylate metabolism	G0Z3A2 (K03781)
ko01200	Carbon metabolism	G0Z3A2 (K03781)
ko03040	Spliceosome	A0A0H5BIT5 (K03283)
ko04010	MAPK signaling pathway	A0A0H5BIT5 (K03283)
ko04068	FoxO signaling pathway	G0Z3A2 (K03781)
ko04110	Cell cycle	A0A2R4PCY0 (K06630)
ko04114	Oocyte meiosis	A0A2R4PCY0 (K06630)
ko04141	Protein processing in endoplasmic reticulum	A0A0H5BIT5 (K03283)
ko04144	Endocytosis	A0A0H5BIT5 (K03283)
ko04145	Phagosome	A0A0H5BIT0 (K05692)
ko04146	Peroxisome	V5LTF3 (K04565); G0Z3A2 (K03781)
ko04151	PI3K-Akt signaling pathway	A0A2R4PCY0 (K06630)
ko04210	Apoptosis	A0A0H5BIT0 (K05692)
ko04211	Longevity regulating pathway	G0Z3A2 (K03781)
ko04213	Longevity regulating pathway-multiple species	V5LTF3 (K04565); G0Z3A2 (K03781); A0A0H5BIT5 (K03283)
ko04350	TGF-beta signaling pathway	E7D7Z1 (K04660)
ko04390	Hippo signaling pathway	A0A2R4PCY0 (K06630)
ko04510	Focal adhesion	A0A0H5BIT0 (K05692)
ko04520	Adherens junction	A0A0H5BIT0 (K05692)
ko04530	Tight junction	A0A0H5BIT0 (K05692)
ko04722	Neurotrophin signaling pathway	A0A2R4PCY0 (K06630)
ko04810	Regulation of actin cytoskeleton	A0A0H5BIT0 (K05692)
ko04918	Thyroid hormone synthesis	X2GM95 (K16141);A0A2S1M4Y6 (K16141)
ko04974	Protein digestion and absorption	A4GIN2 (K19479)
ko05014	Amyotrophic lateral sclerosis (ALS)	V5LTF3 (K04565); G0Z3A2 (K03781)
ko05016	Huntington disease	V5LTF3 (K04565)
ko05020	Prion diseases	V5LTF3 (K04565)
ko05132	Salmonella infection	A0A0H5BIT0 (K05692)
ko05143	African trypanosomiasis	A0A220IG97 (K13823)
ko05144	Malaria	A0A220IG97 (K13823)
ko05164	Influenza A	A0A0H5BIT0 (K05692)
ko05169	Epstein-Barr virus infection	A0A2R4PCY0 (K06630)
ko05203	Viral carcinogenesis	A0A2R4PCY0 (K06630)
ko05205	Proteoglycans in cancer	E7D7Z1 (K04660)

### Renal function and pathology damages

To investigate whether SDAP1 and SDAP2 can improve GM-induced nephrotoxicity in vivo, we established a mouse model of acute gentamicin nephrotoxicity. As shown in Table 5, compared with the control group, GM significantly reduced the weight of mice and significantly increased the kidney index. In contrast to the GM model group, SDAP1 and SDAP2 pre-protection groups partially increased body weight and reduced GM-induced increase in renal index (*P* < 0.01).

**Table 5:** SDAP1 and SDAP2 against GM-induced kidney dysfunction.

	Initial weight (g)	Final weight (g)	Change of weight (g)	Kidney index
Control	30.770 ± 1.52	34.562 ± 1.28	3.762 ± 0.80	0.58 ± 0.05
GM (100 mg/kg)	31.676 ± 1.81	29.225 ± 1.31	− 2.451 ± 0.61**	1.12 ± 0.03**
GM + SDAP1 (15 mg/kg)	30.376 ± 1.62	29.837 ± 1.22	− 0.539 ± 1.08^##^	0.87 ± 0.03^##^
GM + SDAP1 (60 mg/kg)	30.311 ± 1.26	33.773 ± 1.09	3.462 ± 0.72^##^	0.68 ± 0.04^##^
GM + SDAP2 (15 mg/kg)	30.434 ± 1.14	29.616 ± 1.58	− 0.818 ± 0.63^##^	0.94 ± 0.04^##^
GM + SDAP2 (60 mg/kg)	30.805 ± 1.08	33.915 ± 1.16	3.110 ± 0.84^##^	0.73 ± 0.03^##^

Kidney index = (kidney weight (g)/body weight (g)) × 100. Values are expressed as means ± SD (n = 5).

**P < 0.05 vs. Control group.

^##^*P* < 0.01 vs. GM group.

**Figure 6:**
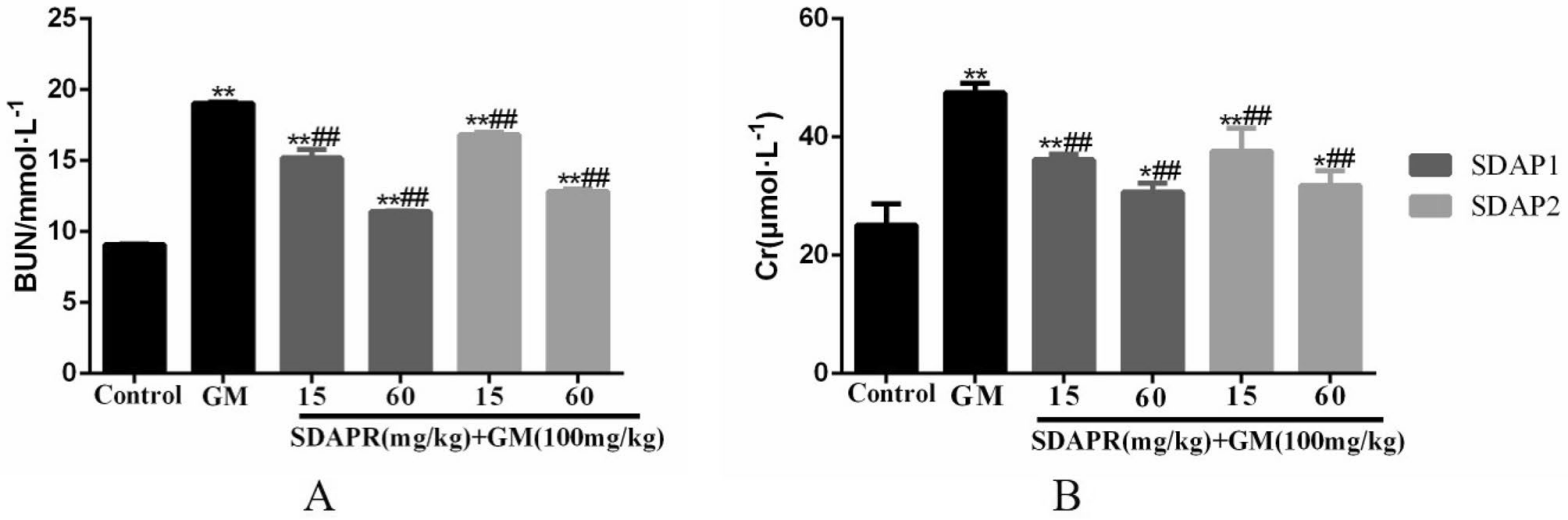
Effects of SDAP1 and SDAP2 treatment on GM-induced serum nephrotoxicity biomarkers in mice. Mice were pretreated with SDAP1 (15, 60 mg/kg) and SDAP2 (15, 60 mg/kg) with or without GM (100 mg/kg) for 24 h. (**A**) BUN levels in serum. (**B**) Cr levels in serum. Data are presented as the Mean ± SD (n = 5). **P < 0.01 versus control group. ^#^*P* < 0.05, ^##^*P* < 0.01 versus GM group.

As shown in Fig. 6A and B, compared with control mice, the levels of renal function indicators (Cr and BUN) in the serum of model mice were significantly increased (*P* < 0.01). Compared with model mice, it was found that SDAP1 and SDAP2 at low and high doses can significantly reduce Cr content, and SDAP1 and SDAP2 at high doses have significant effects. After the detection of BUN content, it was found that low and high doses of SDAP1 and SDAP2 can significantly reduce the content of BUN, and the effects of high doses of SDAP1 and SDAP2 were significant (*P* < 0.01).

**Figure 7:**
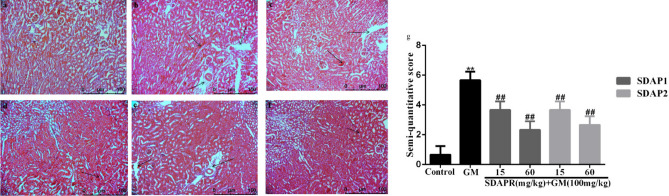
Effect of SDAP1 and SDAP2 treatment on GM-induced histopathology change in mice. (**a**) Control; (**b**) GM (100 mg/kg); (**c**) SDAP1 (15 mg/kg) + GM (100 mg/kg); (**d**) SDAP1 (60 mg/kg) + GM (100 mg/kg); (**e**) SDAP2 (15 mg/kg) + GM (100 mg/kg); (**f**) SDAP2 (60 mg/kg) + GM (100 mg/kg); (**g**) mouse kidney tissue semi-quantitative score. Images are shown at a magnification of 200 × . Data are mean ± SD (n = 3). **P < 0.01 as compared to the Control group. ^##^*P* < 0.01 as compared to the GM-treated group.

The HE staining of the kidneys revealed that the kidney tissue of mice in the control group had a more normal appearance. In the model group, there was obvious edema in the interstitial space of the kidney, severe glomerular shrinkage, and obstruction of the renal tubular cavity, and some cells were swollen and vacuolated. In the SDAP1 and SDAP2 low- and high-dose groups, the renal tubular tissue showed reduced focal lesions, renal tubular swelling and vacuole degeneration were also improved, and inflammatory cell infiltration and interstitial edema were alleviated to varying degrees. SDAP1 and SDAP2 improved better at high doses. The results are shown in Fig. 7.

Semi quantitative analysis of renal tubulointerstitial injury score: inflammatory cell infiltration < 25% is 1 score, 25–49% is 2 score, 50–75% is 3 score, and > 75% is 4 score; renal interstitial edema is 1 score of mild, 2 score of severe; renal tubulointerstitial injury is only 1 score of epithelial cell vacuole degeneration, 2 score of brush border, 3 score of necrosis. The semi quantitative renal tubulointerstitial score is the sum of the above scores (Fig. 7g).

### Oxidative stress and antioxidant defense

**Figure 8:**
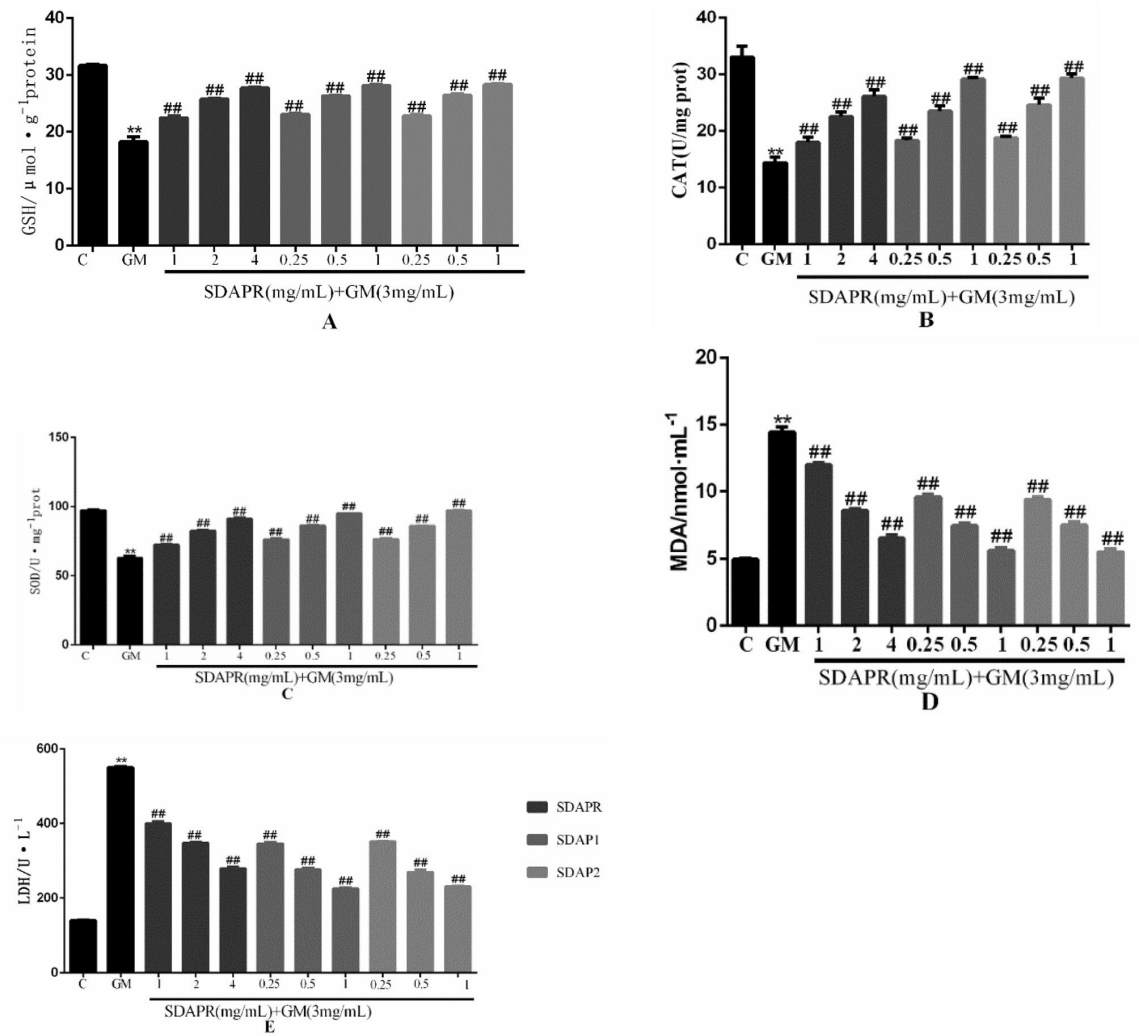
Effect of SDAPR, SDAP1 and SDAP2 treatment on GM-induced the oxidative stress parameters in HEK 293. (**A**) GSH levels in HEK293. (**B**) CAT activity in HEK293. (**C**) SOD activity in HEK293. (**D**) MDA levels in HEK293. (**E**) LDH content in HEK293. Data are means ± SD (n = 4). **P < 0.01 as compared to the Control group. ^##^*P* < 0.01 as compared to the GM-treated group.

**Figure 9:**
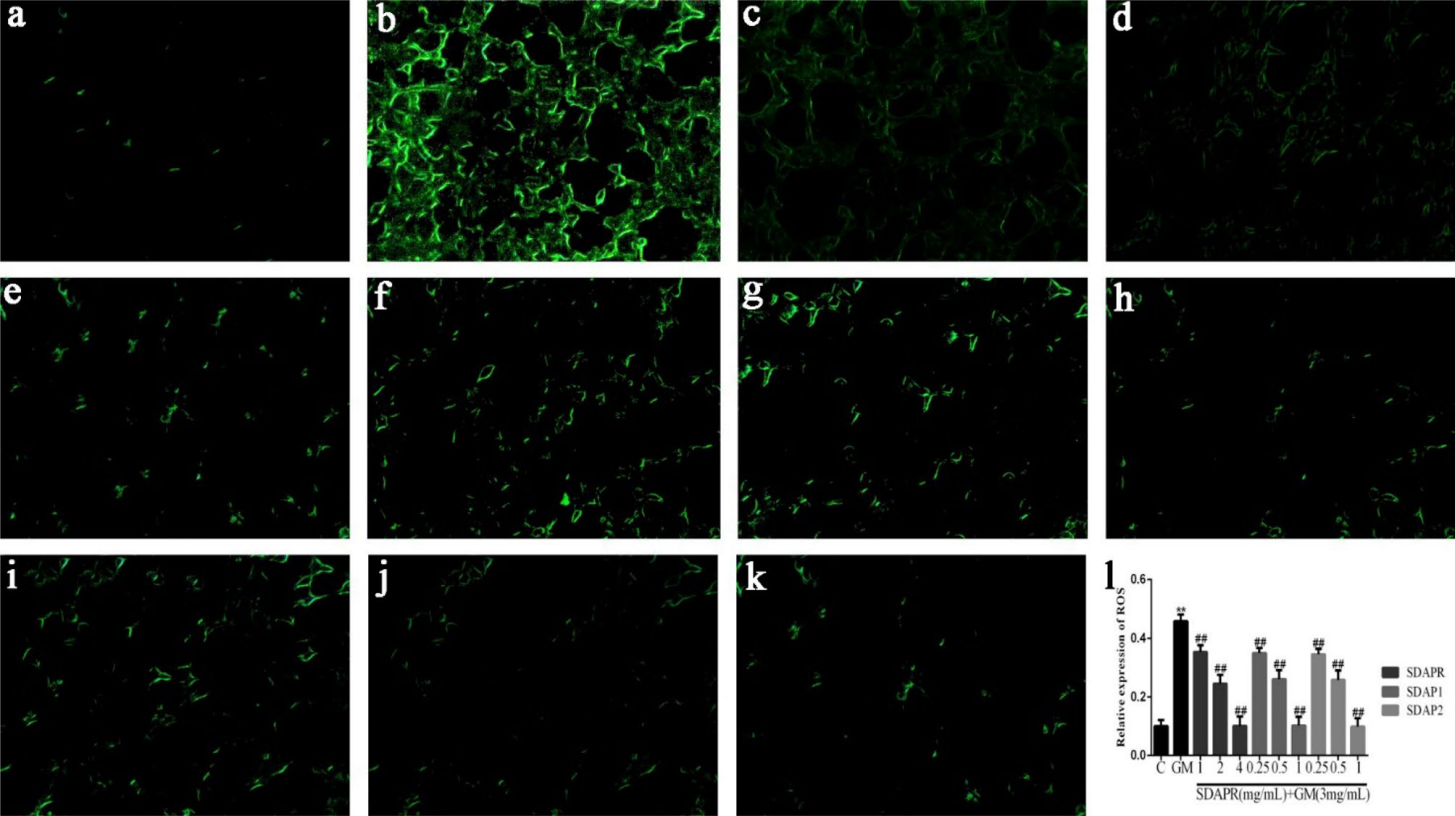
SDAPR, SDAP1 and SDAP2 significantly suppresses ROS production of HEK‑293 cells induced with GM. (**A**) Control; (**B**) GM (3 mg/mL); (**C**) SDAPR (1 mg/mL) + GM (3 mg/mL); (**D**) SDAPR (2 mg/mL) + GM (3 mg/mL); (**E**) SDAPR (4 mg/mL) + GM (3 mg/mL); (**F**) SDAPR (0.25 mg/mL) + GM (3 mg/mL); (**G**) SDAPR (0.5 mg/mL) + GM (3 mg/mL); (**H**) SDAPR (1 mg/mL) + GM (3 mg/mL); (**I**) SDAPR (0.25 mg/mL) + GM (3 mg/mL); (**J**) SDAPR (0.5 mg/mL) + GM (3 mg/mL); (**K**) SDAPR (1 mg/mL) + GM (3 mg/mL); (**L**) Relative expression of ROS in HEK293 cells. Data are means ± SD (n = 3). **P < 0.01 as compared to the Control group. ^##^*P* < 0.01 as compared to the GM-treated group.

In HEK293 cells, GM decreased the level of antioxidants such as GSH (Fig. 8A), CAT (Fig. 8B) and SOD (Fig. 8C), while increased the production of MDA (Fig. 8D) and LDH (Fig. 8E). Conversely, SDAPR (1, 2, 4 mg/mL), SDAP1 and SDAP2 (0.25, 0.5, 1 mg/mL) reversed the decrease in HEK293 cells SOD, GSH and GSH, and the overproduction of MDA and LDH. Excessive ROS induced by harmful stimulation will cause oxidative damage to biomolecules (such as lipids, DNA, proteins, etc.), thus inducing cell injury or death. The changes in intracellular ROS were detected by DCFH-DA staining. Damaged cells exerted strong fluorescent intensity, which indicated that massive ROS generated. HEK-293 cells were treated with GM and SDAPR, SDAP1, SDAP2 for 24 h and ROS level was detected using the ROS fluorescent probe DCFH-DA. As shown in Fig. 9, SDAPR, SDAP1 and SDAP2 treatment obviously decreased GM-induced intracellular ROS accumulation.

### Mitochondrial dysfunction and apoptosis

**Figure 10:**
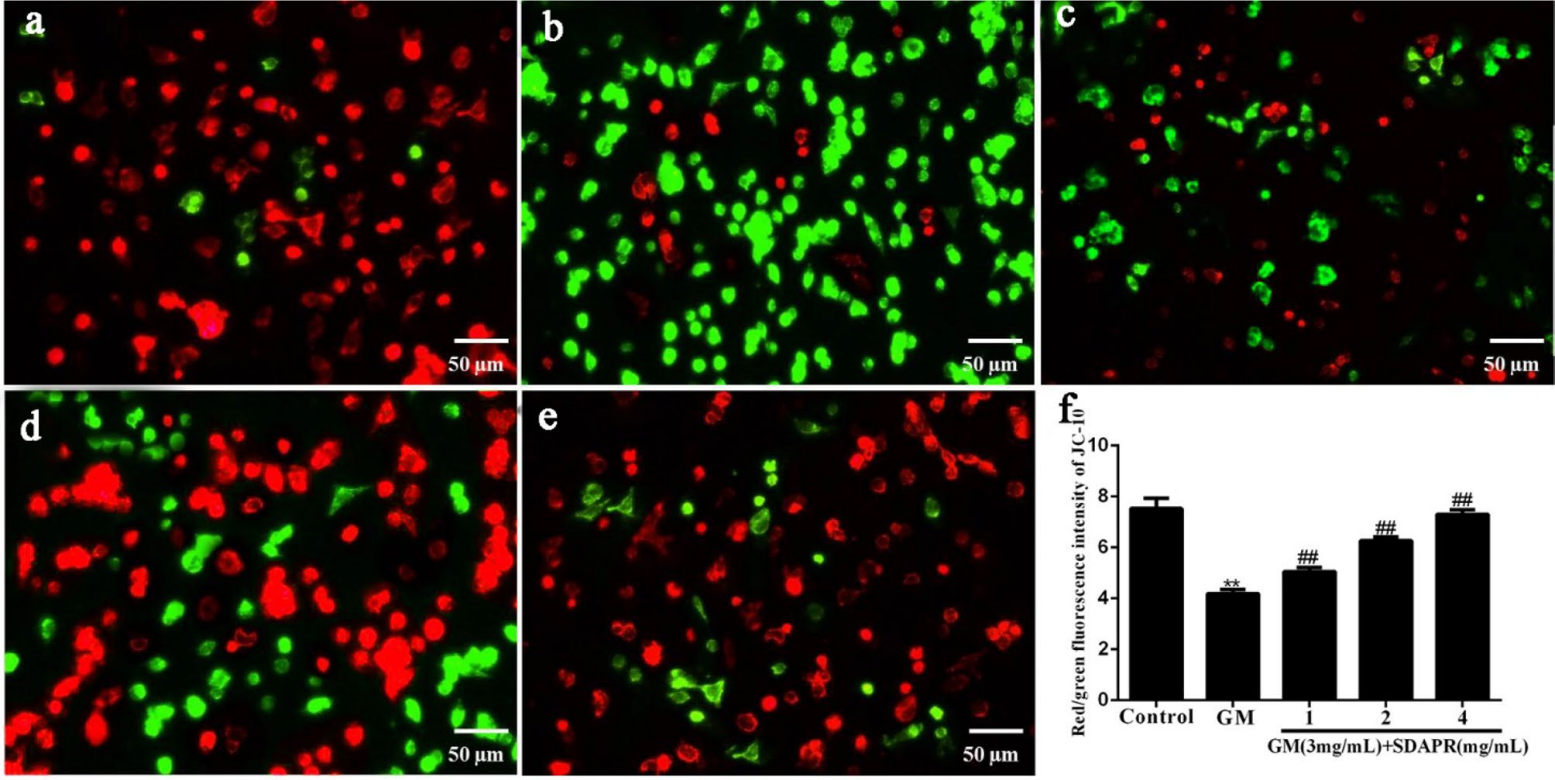
Effects of SDAPR on GM induced mitochondrial membrane potential (ΔΨm) depolarization in HEK-293 cells (scalebar, 50 μm). (**A**) Control; (**B**) GM (3 mg/mL); (**C**) SDAPR (1 mg/mL) + GM (3 mg/mL); (**D**) SDAPR (2 mg/mL) + GM (3 mg/mL); (**E**) SDAPR (4 mg/mL) + GM (3 mg/mL); (**F**) Red/green fluorescence intensity of JC-10 in HEK293 cells. Data are presented as the Mean ± SD (n = 6). **P < 0.01 versus control group. ^##^*P* < 0.01 versus GM group. *SDAPR* Sika deer antler protein, *GM* gentamicin, *HEK293* human embryonic kidney 293.

In order to maintain mitochondrial function, balancing mitochondrial membrane potential is critical. Compare with the control group, HEK293 cells of GM group reduced the production red fluorescence meanwhile increased the intensity of green fluorescence (*P* < 0.01). By contrast, SDAPR (1.0, 2.0 and 4.0 mg/mL) significantly increased the red/green fluorescence ratio of HEK293 cells which pretreated with SDAPR (*P* < 0.01 and *P* < 0.01, respectively). Our data suggested that SDAPR pretreatment reverses mitochondrial depolarization and maintains mitochondrial function (Fig. 10).

**Figure 11:**
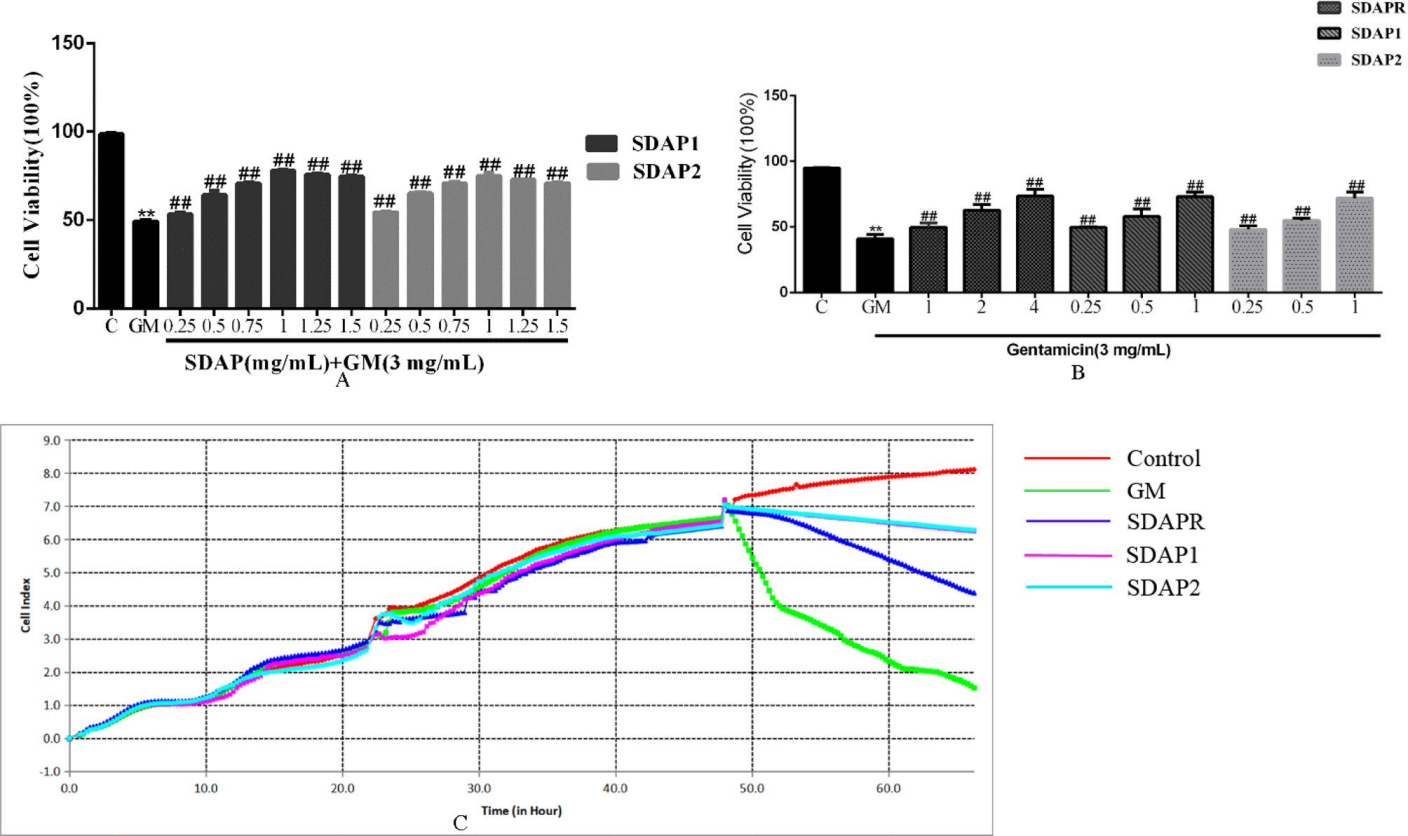
Effect of different components of SDAPR on GM-induced cytotoxicity in HEK-293 cells. (**A**) Cells were pretreated with SDAP1 and SDAP2 (0.25, 0.5, 0.75, 1, 1.25, 1.5 mg/mL) before administration of GM (3 mg/mL) for 24 h. Data are means ± SD (n = 6). (**B**) Cells were pretreated with SDAPR (1, 2, 4 mg/mL), SDAP1 and SDAP2 (0.25, 0.5, 1 mg/mL) before administration of GM (3 mg/mL) for 24 h. Data are means ± SD (n = 6). **P < 0.01 as compared to the Control group. ^##^*P* < 0.01 as compared to the GM-treated group. (**C**) Cells were pretreated with SDAPR (4 mg/mL), SDAP1 and SDAP2 (1 mg/mL) before administration of GM (3 mg/mL) for 24 h. Data are means ± SD (n = 3). **P < 0.01 as compared to the Control group. ^##^*P* < 0.01 as compared to the GM-treated group.

MTT and RTCA experiments were performed to study the effects of SDAPR, SDAP1 and SDAP2 on the viability of GM-induced HEK293 cells. To screen the pre-protection of SDAP1 and SDAP2, cells were pretreated with SDAP1 and SDAP2 (0.25, 0.5, 0.75, 1, 1.25, 1.5 mg/mL) before administration of GM (3 mg/mL) for 24 h (Fig. 11A). We selected SDAP1 and SDAP2 at 0.25, 0.5, and 1 mg/mL for subsequent experiments. Compared with the control group, GM (3 mg/mL) significantly reduced cell viability. In addition, the other groups pretreated with SDAPR (1, 2, 4 mg/mL), SDAP1 (0.25, 0.5, 1 mg/mL), and SDAP2 (0.25, 0.5, 1 mg/mL) for 24 h had significant differences compared with the GM group (*P* < 0.01). These results indicate that SDAPR, SDAP1 and SDAP2 provide dose-dependent protection against GM-induced cell damage (Fig. 11B). As shown in Fig. 11C, RTCA experiments confirmed that SDAP1 (1 mg/mL) and SDAP2 (1 mg/mL) were more protective than SDAPR (4 mg/mL).

**Figure 12:**
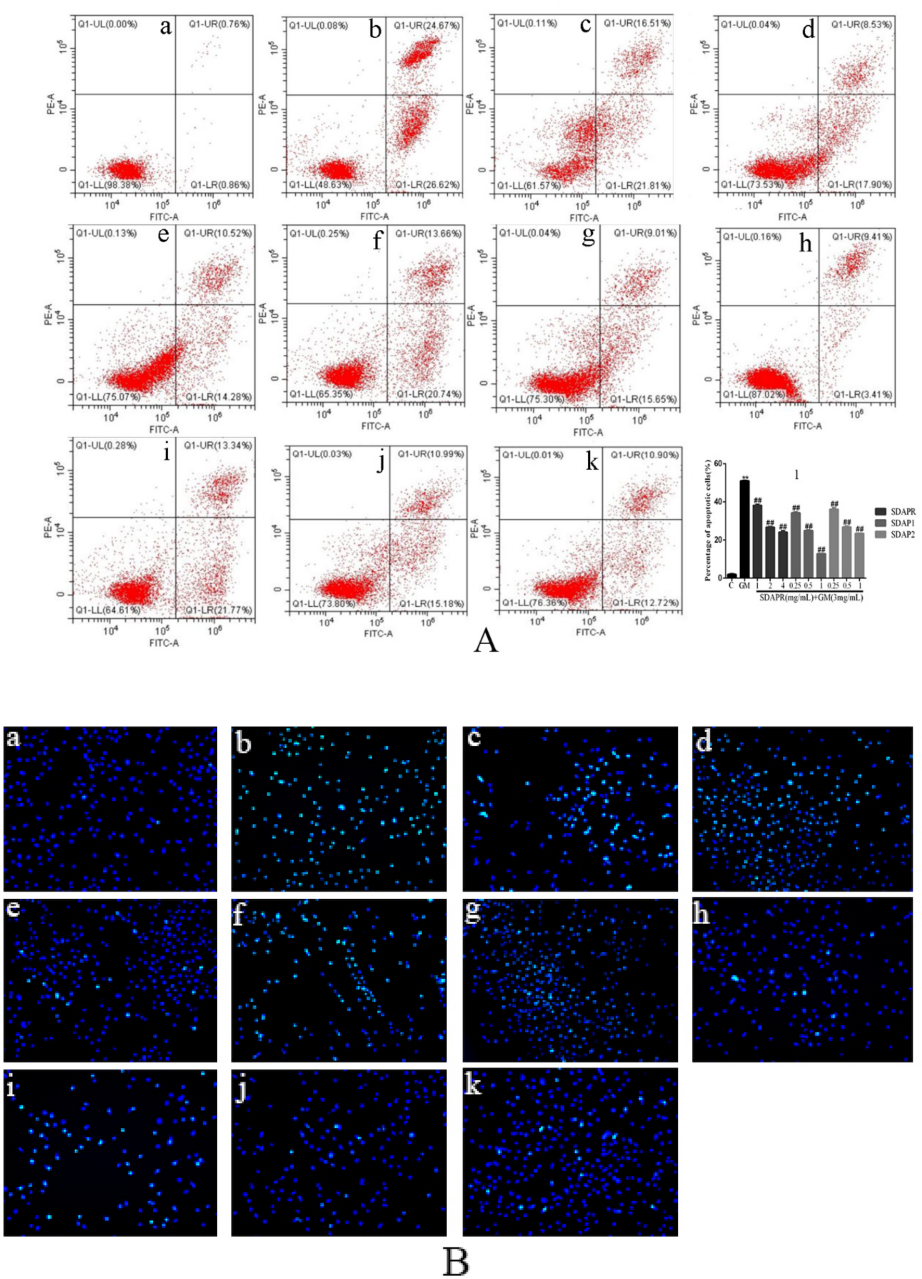
Effect of SDAPR, SDAP1 and SDAP2 on apoptosis induction in GM treated HEK-293 cells. (**A**) Result of the apoptosis in HEK293 cells were analyzed by flow cytometry. (**B**) The nuclear morphology of HEK293 cells were evaluated using Hoechst 33,258 staining. (a) Control; (b) GM (3 mg/mL); (c) SDAPR (1 mg/mL) + GM (3 mg/mL); (d) SDAPR (2 mg/mL) + GM (3 mg/mL); (e) SDAPR (4 mg/mL) + GM (3 mg/mL); (f) SDAPR (0.25 mg/mL) + GM (3 mg/mL); (g) SDAPR (0.5 mg/mL) + GM (3 mg/mL); (h) SDAPR (1 mg/mL) + GM (3 mg/mL); (i) SDAPR (0.25 mg/mL) + GM (3 mg/mL); (j) SDAPR (0.5 mg/mL) + GM (3 mg/mL); (k) SDAPR (1 mg/mL) + GM (3 mg/mL). Data are presented as the Mean ± SD (n = 3). **P < 0.01 versus control group. ^#^*P* < 0.05, ^##^*P* < 0.01 versus GM group.

**Figure 13:**
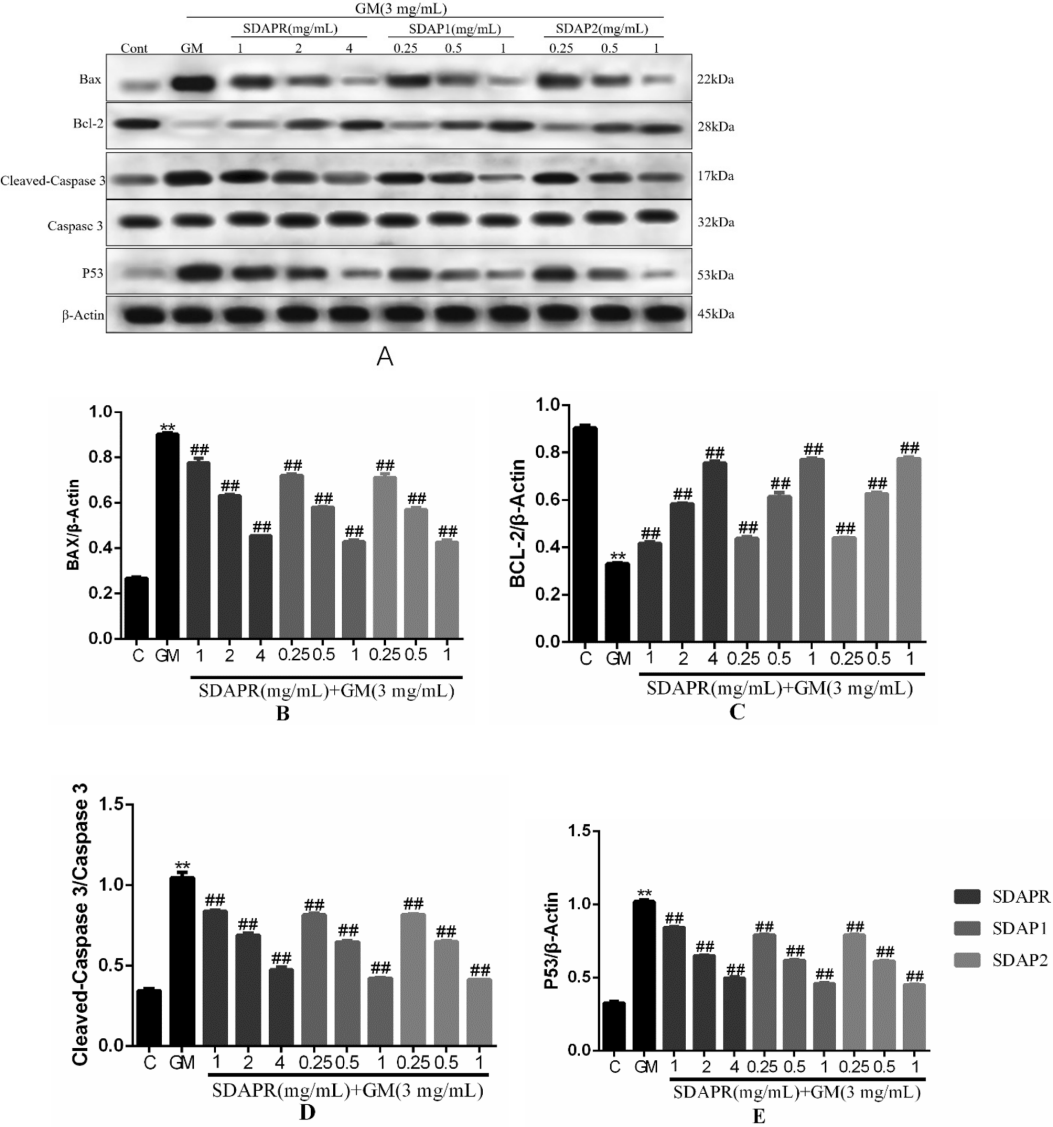
Detection of Bax, Bcl-2, Cleaved-Caspase 3 and P53 protein expression by Western blotting. (**A**) Immunoblotting for the target molecule of Bax, Bcl-2, Cleaved-Caspase 3 and Caspase 3 showing the effects of SDAPR, SDAP1 and SDAP2 in GM treated HEK-293 cells; (**B**) expression of Bax was determined by western blotting; (**C**) expression of Bcl-2 was determined by western blotting; (**D**) expression of Cleaved-caspase 3 was determined by western blotting; (**E**) expression of P53 was determined by western blotting. Data are means ± SD (n = 3). **P < 0.01 as compared to the Control group. ^##^*P* < 0.01 as compared to the GM-treated group.

In order to investigate whether GM-mediated toxicity of HEK-293 cells is related to the activation of apoptotic cells, flow cytometry was used to detect apoptotic cells. As shown in Fig. 12, compared with the control group, the total apoptotic cells (early and late apoptotic) increased significantly to 51.29% (*P* < 0.01) in the GM treatment group of 3 mg/mL for 24 h. However, after pretreatment with SDAPR (1 mg/mL, 2 mg/mL and 4 mg/mL), the apoptotic cells decreased from 51.29 to 38.32%, 26.43% and 24.80% respectively. Moreover, after SDAP1 (0.25 mg/mL, 0.5 mg/mL and 1 mg/mL) pretreatment, the apoptotic cells decreased from 51.29 to 34.40%, 24.66% and 12.82% respectively. At the same time, SDAP2 (0.25 mg/mL, 0.5 mg/mL and 1 mg/mL) was used to protect the apoptotic cells from 51.29 to 35.11%, 26.17% and 23.62% respectively. The results showed that although GM treatment resulted in increased apoptosis, the combination of SDAPR, SDAP1 and SDAP2 had the opposite effect on GM-induced apoptosis (Fig. 12A). In addition, we also used Hoechst 33258 staining to determine the effects of SDAPR, SDAP1 and SDAP2 on GM-induced apoptosis of HEK-293 cells. In Fig. 13B, the results showed that compared with the control group, Hoechst 33258 staining positive cells of HEK293 cells in GM group increased, and the morphology of typical apoptotic cells such as chromatin agglutination (bright blue fluorescence), fragmentation and marginalization increased. Compared with GM group, the Hoechst33258 staining positive cells of HEK293 cells in the SDAPR, SDAP1 and SDAP2 administration groups decreased, and the typical apoptotic cell morphology such as chromatin condensation (bright blue fluorescence), fragmentation and marginalization was alleviated with increasing drug concentration (Fig. 12B).

**Figure 14:**
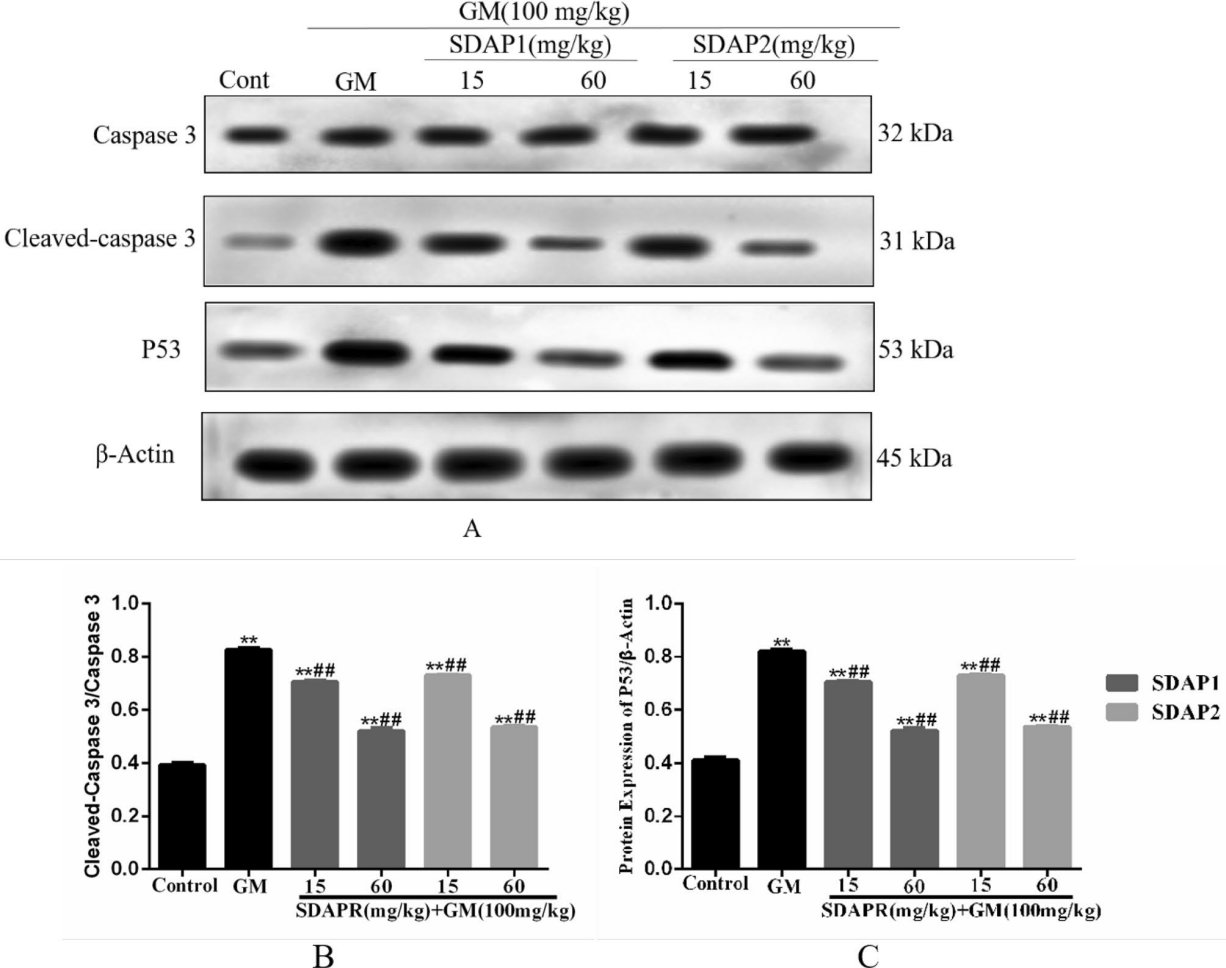
Western blot was used to detect the expression of Cleaved-Caspase 3 and P53 protein. (**A**) Western blot of Cleaved-Caspase 3 and P53 target molecules, showing the role of SDAP1 and SDAP2 in kidneys of GM-treated mice; (**B**) Western blot to determine the expression of Cleaved-caspase 3; (**C**) Western blot to determine the expression of P53 expression. Data are mean ± SD (n = 3). **P < 0.01 as compared to the Control group. ^##^*P* < 0.01 as compared to the GM-treated group.

At the same time, we used Western blot analysis to detect the expression of apoptosis-related proteins in vivo and in vitro. The results showed that treatment of HEK293 cells with GM reduced the expression of the anti-apoptotic protein BCL-2, and the administration of SDAPR, SDAP1 and SDAP2 significantly decreased the expression of BCL-2. We also evaluated the expression of pro-apoptotic Bax and found that its expression was significantly increased after GM treatment (*P* < 0.01), however, with the addition of SDAPR, SDAP1 and SDAPR significantly reduced Bax expression. At the same time, the expression of cleaved-caspase-3 was also evaluated. Western blot analysis showed that SDAPR, SDAP1 and SDAP2 could reverse the increase of GM-induced cleaved-caspase-3 protein expression in HEK293 cells. In addition, p53 expression was also increased after GM treatment compared with the control group (*P* < 0.01). SDAPR, SDAP1 and SDAP2 attenuate GM-induced increase in p53 expression (Fig. 13). In vivo, we evaluated the expression of cleaved-Caspase-3 using Western blot analysis (Fig. 14). Western blot analysis showed that SDAP1 and SDAP2 can reverse the increase in Caspase-3 cleavage protein expression. In addition, compared with the control group, p53 expression was also increased after GM treatment (*P* < 0.01). SDAP1 and SDAP2 attenuated the GM-induced increase in p53 expression.

### Nrf2, NF-κB and PI3K analysis

**Figure 15:**
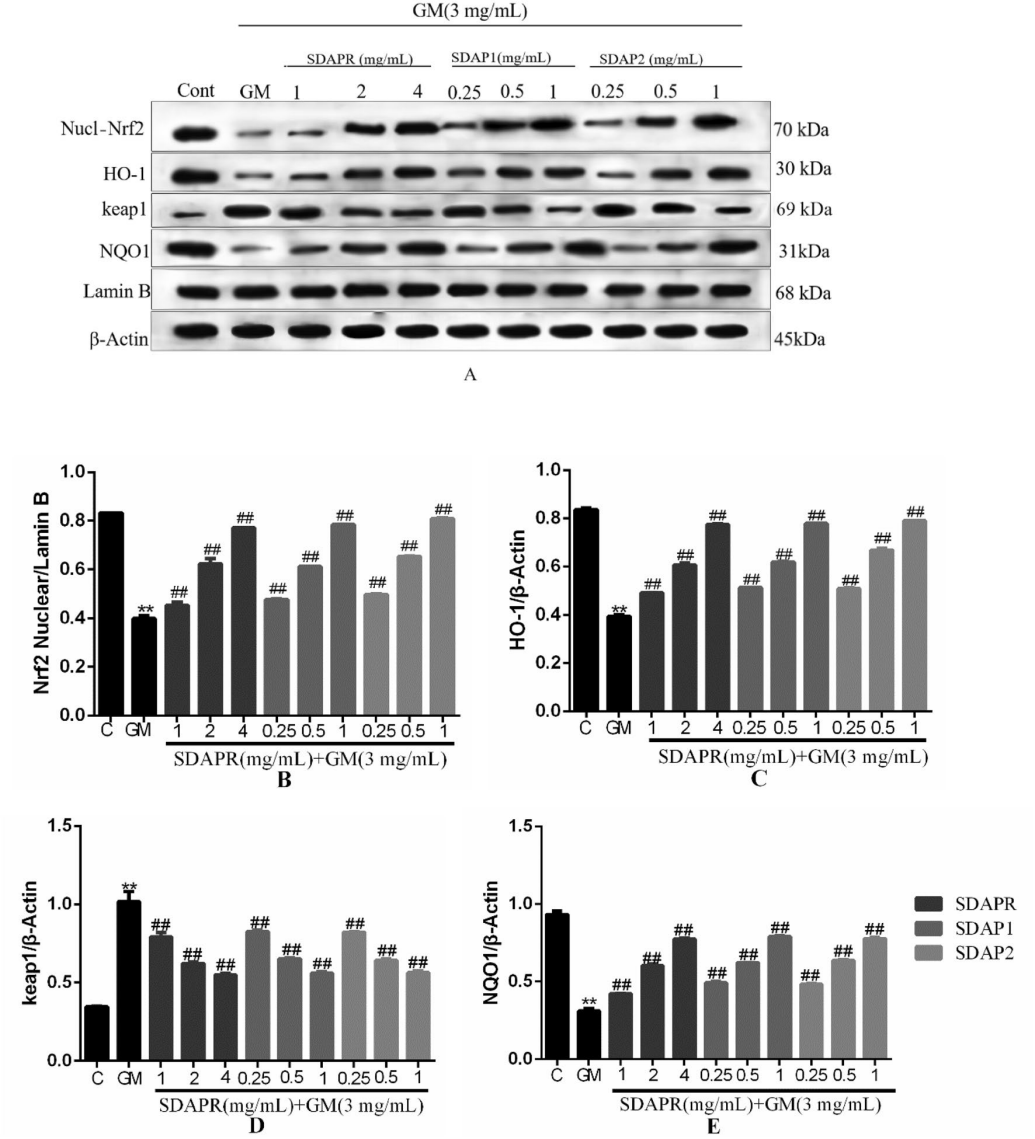
Detection of Nrf2, HO-1, keap1, NQO1 protein expression by Western blotting. (**A**) Immunoblotting for the target molecule of Nrf2, HO-1, keap1 and NQO1 showing the effects of SDAPR, SDAP1 and SDAP2 in GM treated HEK-293 cells; (**B**) expression of Nrf2 was determined by western blotting; (**C**) expression of HO-1 was determined by western blotting; (**D**) expression of keap1 was determined by western blotting; (**E**) expression of NQO1 was determined by western blotting. Data are means ± SD (n = 3). **P < 0.01 as compared to the Control group. ^##^*P* < 0.01 as compared to the GM-treated group.

**Figure 16:**
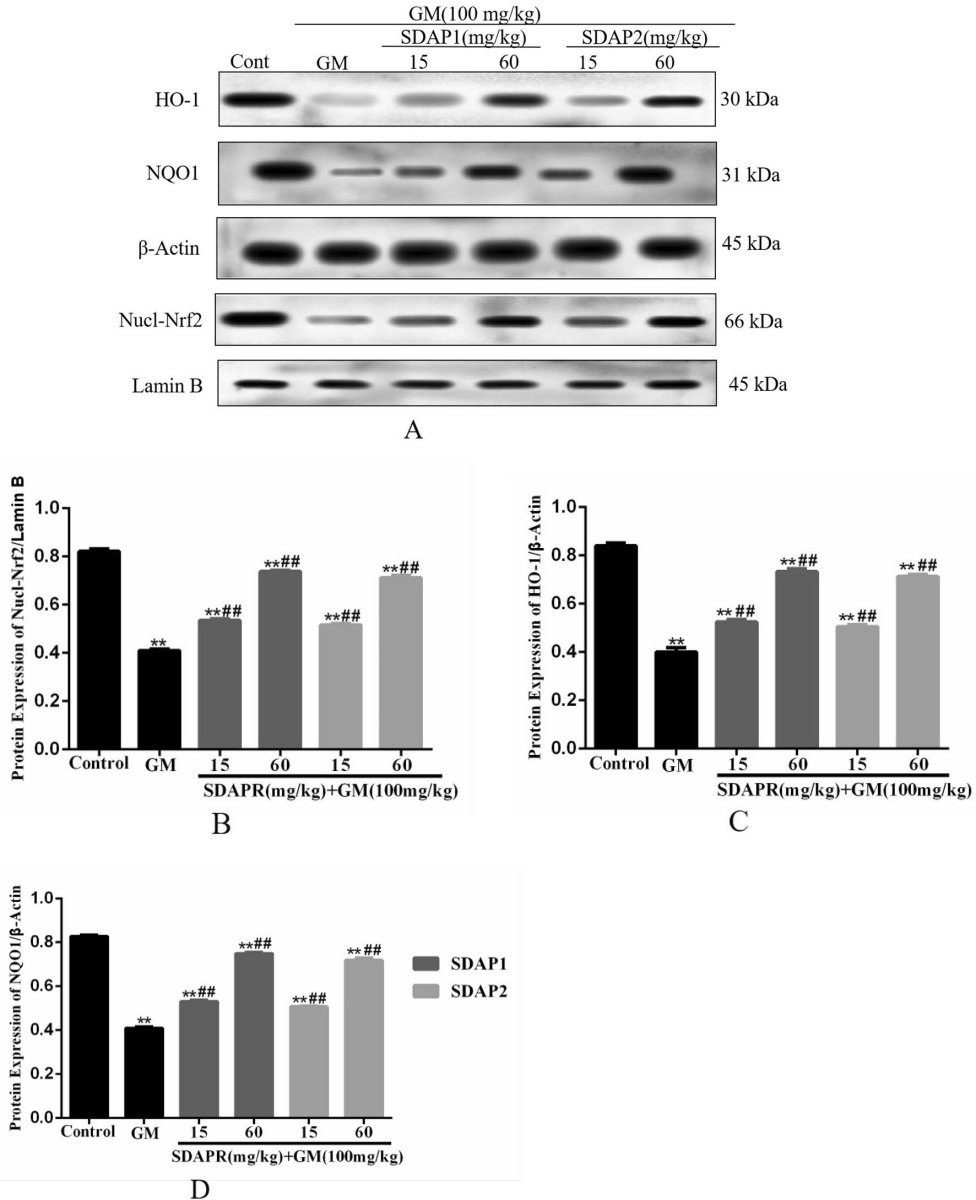
Western blot was used to detect the expression of Nrf2 pathway protein. (**A**) Western blot of Nrf2, HO-1 and NQO1; (**B**) Western blot to determine the expression of Nrf2; (**C**) Western blot to determine the expression of HO-1; (**D**) Western blot to determine the expression of NQO1. Data represent mean ± SD (n = 3). **P < 0.01 as compared to the Control group. ^##^*P* < 0.01 as compared to the GM-treated group.

To investigate the molecular basis of a protective effect of SDAPR against GM-induced HEK293 cells damage. Figure 15A is a Western blot of nuclear Nrf2, total HO-1, total keap1 and total NQO1. We examined whether SDAPR, SDAP1 and SDAP2 treatment affected HEK293 cells expression of nuclear Nrf2 (Fig. 15B), total HO-1 (Fig. 15C), total keap1 (Fig. 15D) and total NQO1 (Fig. 15E). GM markedly reduced expression of nuclear Nrf2, total HO-1, and total NQO1 (*P* < 0.01). However, compared with the group receiving only GM, SDAPR, SDAP1 and SDAP2 significantly reversed these effects (*P* < 0.01). In addition, the expression of total Keap1 protein was significantly increased in GM group, which was reversed by SDAPR, SDAP1 and SDAP2 (*P* < 0.01). Figure 16A is a Western blot of nuclear Nrf2, total HO-1 and total NQO1 in vivo. At the same time, it was investigated whether SDAP1 and SDAP2 treatment affected the expression of Nrf2 (Fig. 16B), total HO-1 (Fig. 16C), and total NQO1 (Fig. 16D) in the renal nucleus of mice. GM significantly reduced the expression of nuclear Nrf2, total HO-1 and total NQO1 (*P* < 0.01). However, SDAP1 and SDAP2 significantly reversed these effects compared to the group receiving only GM (*P* < 0.01).

**Figure 17:**
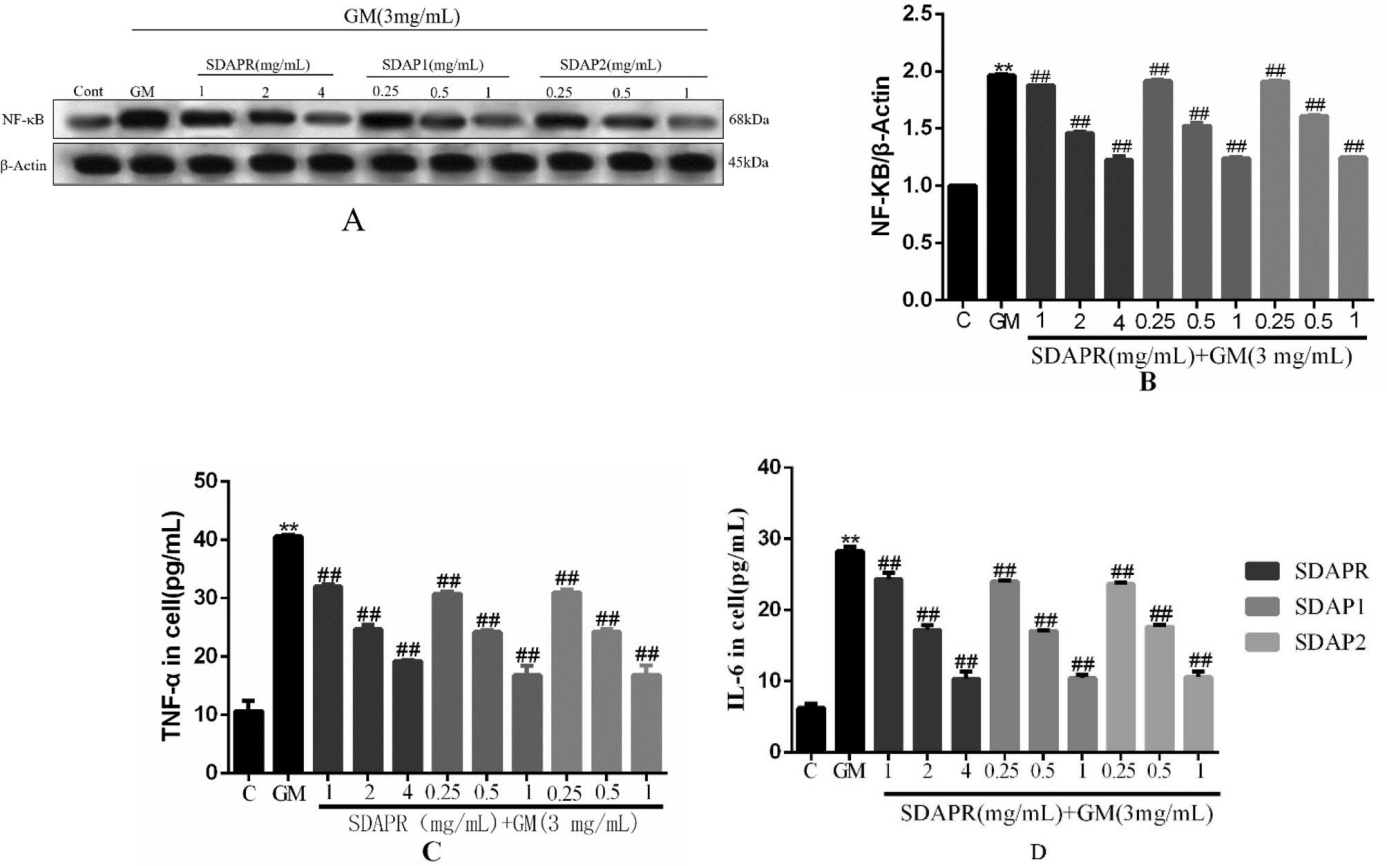
Determination of inflammation index in HEK293 cells. (**A**) Immunoblotting for the target molecule of NF-κB showing the effects of SDAPR, SDAP1 and SDAP2 in GM treated HEK-293 cells; (**B**) expression of NF-κB was determined by western blotting; (**C**) Elisa assay for TNF-α in HEK293 cells; (**D**) Elisa assay for IL-6 in HEK293 cells. Data are means ± SD (n = 3). **P < 0.01 as compared to the Control group. ^##^*P* < 0.01 as compared to the GM-treated group.

Here, we validated the effect of SDAPR, SDAP1 and SDAP2 on TNF-α, IL-6 and NF-κB in GM-induced inflammation. The results showed that the production of inflammatory cytokines TNF-α and IL-6 increased in the GM group, while the production of TNF-α and IL-6 in the SDAPR, SDAP1 and SDAP2 treatment groups decreased (*P* < 0.01). In addition, SDAPR, SDAP1 and SDAP2 also significantly reduced GM-induced NF-κB expression in HEK293 cells (Fig. 17).

**Figure 18:**
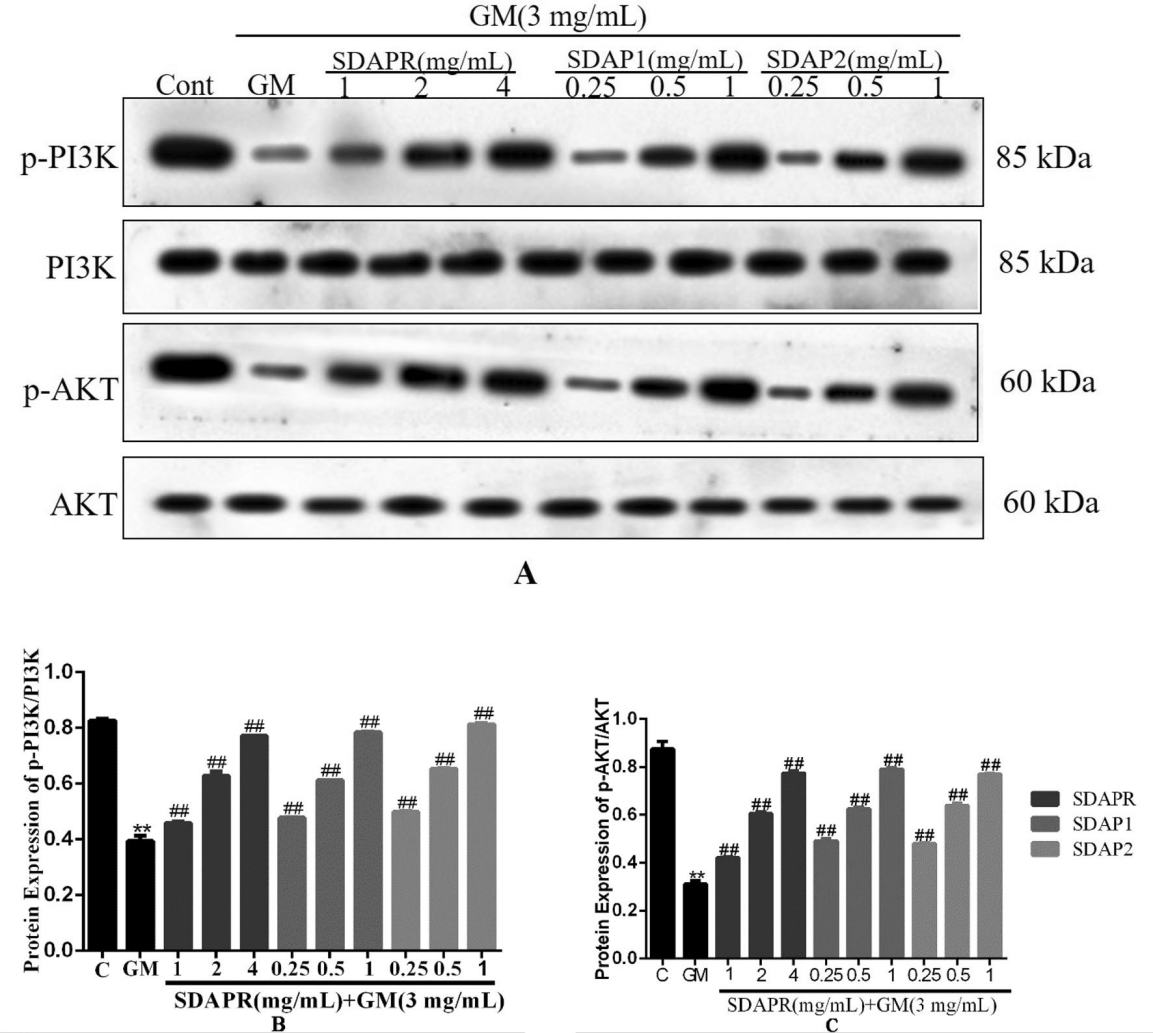
Detection of PI3K, p-PI3K, AKT, p-AKT protein expression by Western blotting. (**A**) Immunoblotting for the target molecule of PI3K, p-PI3K, AKT, and p-AKT showing the effects of SDAPR, SDAP1 and SDAP2 in GM treated HEK-293 cells; (**B**) expression of p-PI3K/PI3K was determined by western blotting; (**C**) expression of p-AKT/AKT was determined by western blotting. Data are means ± SD (n = 3). **P < 0.01 as compared to the Control group. ^##^*P* < 0.01 as compared to the GM-treated group.

We hypothesized that SDAPR, SDAP1 and SDAP2 against GM-induced oxidative stress and apoptosis effects may be related to PI3K/AKT Pathway by KEGG analysis (Fig. 4B). Therefore, we examined the expression of PI3K, p-PI3K, Akt, p-Akt, in each group of HEK293 cells by Western blot (Fig. 18). Compared with the control group, the expression of p-PI3K and p-Akt were significantly lower in the GM model group (*P* < 0.01). SDAPR (1, 2, 4 mg/mL), SDAP1 (0.25, 0.5, 1 mg/mL) and SDAP2 (0.25, 0.5, 1 mg/mL) pretreatment dose-dependently increased the phosphorylation of PI3K and Akt (P < 0.01).

## Discussion

The clinical application of gentamicin is seriously limited by its ototoxic nephrotoxicity, which leads to the decrease of its antibacterial activity^21^. Although the specific mechanism of GM-induced nephrotoxicity remains unclear, there is increasing evidence that oxidative stress, inflammatory response and cell apoptosis play an important role in GM-induced nephrotoxicity^22^. Antioxidant therapy can help prevent this nephrotoxic effect^23^. The protein content of Sika deer antler is high (51% to 57%), which is considered to be the active ingredient to play a pharmacological role^24^. In addition, protein is the main carrier of life activities and plays a vital role in the smooth progress of various life processes. Therefore, the study of active proteins in pilose antler will be helpful to prevent nephrotoxicity and elucidate its molecular mechanism.

In a mouse model, we demonstrated that co-administration of SDAP can reduce GM-induced nephrotoxicity, as shown by the reduction in BUN, serum creatinine, and caspase-dependent apoptosis levels (Fig. 6). Increased ROS production is an important mechanism for GM-induced nephrotoxicity. Excessive levels of ROS can damage DNA, lipids and proteins, eventually leading to apoptosis^25^. SDAP has been shown to interact with ROS, thereby preventing ROS-induced tissue and cell damage. Consistent with previous studies, we found that GM-induced nephrotoxicity is significantly associated with increased MDA levels, increased LDH levels and decreased levels of antioxidant enzymes CAT, SOD and GSH^26^. In this study, SDAP treatment restored the levels of all these biomarkers to the normal range observed in the untreated control group (Fig. 8), confirming its protective effect on the kidneys. Histopathological examination of the mouse kidney also confirmed this protection (Fig. 7). Compared with the GM model group, the SDAP-treated group had significantly improved renal tubular necrosis and markedly relieved renal tissue damage. Mitochondria is the main target of oxidative damage caused by ROS^27^. In this study, GM treatment significantly disrupted the mitochondrial membrane potential (Fig. 10) of HEK293 cells, which is an indicative feature of mitochondrial dysfunction. Mitochondrion is the integration of internal and external apoptotic signals, which plays an important role in the initiation of mitochondrion mediated apoptosis^28^. The damaged mitochondrial membrane may affect the opening of the mitochondrial permeability transition pore (MPTP), which then leads to the release of cytochrome c and the activation of caspase-9, -3 and the formation of apoptotic bodies^29^. Caspase-3 is a key biomarker of apoptosis, which can be activated through internal and external apoptosis pathways, leading to DNA breakdown^30^. Apoptosis is mainly regulated by Bcl-2 family proteins, especially pro-apoptotic Bax and anti-apoptotic Bcl-2 proteins^31^. When Bax forms a heterodimer with Bcl-2, the release of cytochrome C from mitochondria and subsequent cell death are prevented^32^. In this study, activation of cleaved-caspase-3 was detected after GM exposure (Fig. 13). And 3 mg/mL GM could significantly increase the protein levels of Bax, and decrease the expression of Bcl-2 (Fig. 13). Interestingly, SDAP treatment significantly attenuated mitochondrial membrane destruction and the activation of cleaved-caspase-3, which subsequently reduced GM-induced apoptosis (Fig. 13). These results indicated that SDAP can reduce GM-induced apoptosis by inhibiting the mitochondrial apoptosis pathway.

Nrf2 is a key transcription factor that regulates antioxidant genes (such as CAT, SOD and HO-1) by combining with antioxidant response elements^33^. It has been shown that the activation of Nrf2 is related to cisplatin, acetaminophen and aristolochic acid in drug-induced nephrotoxicity^34^. Similarly, in HEK293 cells, after SDAP treatment, Nrf2 and its corresponding downstream HO-1, NQO1 protein levels increased significantly (Fig. 15). Consistently, SDAP pretreatment further activated Nrf2 and HO-1 protein expression, increased HO-1 activity, and then inhibited GM induced HEK293 apoptosis (Fig. 15). In addition, activated Nrf2 can induce the increase of GSH levels by inducing the expression of cysteine-glutamic acid exchange transporter, thereby protecting cells from oxidative stress^35^. These indicated that the activation of the Nrf2/HO-1 pathway may partly explain the activity of the antioxidant enzymes SOD and CAT and the SDAP-induced increase in GSH content. In conclusion, the activation of the Nrf2/HO-1 pathway plays a protective role in GM-induced apoptosis, and this activation also mediates the protective effect of SDAP.

NF-κB is a family of transcription factors, also known as major regulators in inflammation, oxidative stress and immunity^36^. NF-κB signaling pathway may be activated or inactivated by different factors or stimulation signals, such as tumor necrosis factor-A, IL-6, Caspase-3, c-Jun N-terminal kinase and ROS^37^. On the contrary, activation of NF-κB can induce the expression of many proinflammatory genes, including COX-2, TNF-α, IL-6 and iNOS^38^. Previous studies have shown that GM can induce severe inflammatory reactions, including activation of NF-κB and iNOS, and the production of TNF-α^39^. In this study, GM exposure significantly increased NF-kB protein expression and increased TNF-α and IL-6 levels (Fig. 17). Consistent with these findings, our results have shown that GM-induced nephrotoxicity is associated with significantly increased levels of NF-kB expression and TNF-α and IL-6 levels.

The Nrf2 and NF-κB systems may have crosstalk during oxidative stress and inflammation^40^. Nrf2 activation can inhibit the NF-κB nuclear translocation-mediated inflammatory response through HO-1 end products bilirubin and CO^40^. In this study, HO-1 activation (Fig. 15) attenuated GM-induced caspase-3 (Fig. 13) activation and NF-κB (Fig. 17) protein expression, and ultimately improved the protective effect of SDAP on GM-induced cytotoxicity. These indicated that Nrf2-mediated HO-1 activation partially contributes to SDAP's ability to inhibit the mitochondrial apoptosis pathway. Related research also shown that inhibiting HO-1 can enhance the expression of NF-κB and aggravate the inflammation induced by lipopolysaccharide (LPS)^41^. Therefore, SDAP can partially inhibit GM-induced inflammation through Nrf2/HO-1 activation and down-regulation of NF-κB expression. The inhibitory effect of SDAP on NF-κB may partially promote the activation of HO-1. Obviously, further research is needed to determine the detailed mechanism.

In short, in our study, SDAPR, SDAP1, and SDAP2 significantly improved GM-induced cytotoxicity of HEK293, mainly by improving inflammatory response, reducing apoptosis and enhancing antioxidant capacity. Further analysis indicated that SDAPR, SDAP1 and SDAP2 may play a chemopreventive role by activating Nrf2 and inhibiting NF-κB signaling pathway.

## Conclusion

In conclusion, the results show that SDAP can relieve GM-induced nephrotoxicity by activating Nrf2 pathway. In addition, we found that SDAPR, SDAP1 and SDAP2 inhibited TNF-α expression, activation of NF-κB pathway and GM-induced inflammation in HEK293 cells. Furthermore, SDAP1 and SDAP2 enhanced Nrf2 activation in kidney tissue of GM-treated mice. In summary, our study suggests that SDAPR, SDAP1 and SDAP2, as natural antioxidants, may be used to treat human renal toxicity induced by GM.

## Supplementary information

Supplementary Information

## Data Availability

All data generated or analyzed during this study are included in this manuscript (and its Supplementary Information file).
