# Supplementary material for: Purified Sika deer antler protein attenuates GM-induced nephrotoxicity by activating Nrf2 pathway and inhibiting NF-κB pathway
Source: Sci Rep. 2020 Sep 24;10:15601. doi: 10.1038/s41598-020-71943-6 (PMC7518274; doi:10.1038/s41598-020-71943-6)

**Supplementary materials corresponding to:**

**Purified Sika deer antler protein attenuates GM-induced nephrotoxicity by activating Nrf2 pathway and inhibiting NF-κB pathway**

Zhenyi Wang ^1^ Lulu Wang ^1,2^ Jing Wang ^3^ Jiacheng Luo ^1^ Haonan Ruan ^1^  Jing Zhang ^1,2^

1. *College of Chinese Medicine Materials, Jilin Agricultural University, Changchun, 130118, China*

2. *Changchun Institute of Technology School of Medicine, Changchun, 130600, China*

3 *Jilin Province FAW General Hospital, Changchun, 130000, China*

*Corresponding author at: College of Chinese Medicine Materials, Jilin Agricultural University, Xincheng road 2888, Changchun, 13-118, E-mail: zhjing0701@163.com (J. Zhang)

**Uncropped Western Blots in HEK293 cells and kidney.** **The manuscript picture corresponds to the cropped image in the original manuscript.** **Repeated experiments 2 and 3 represent the images of repeated experiments.**


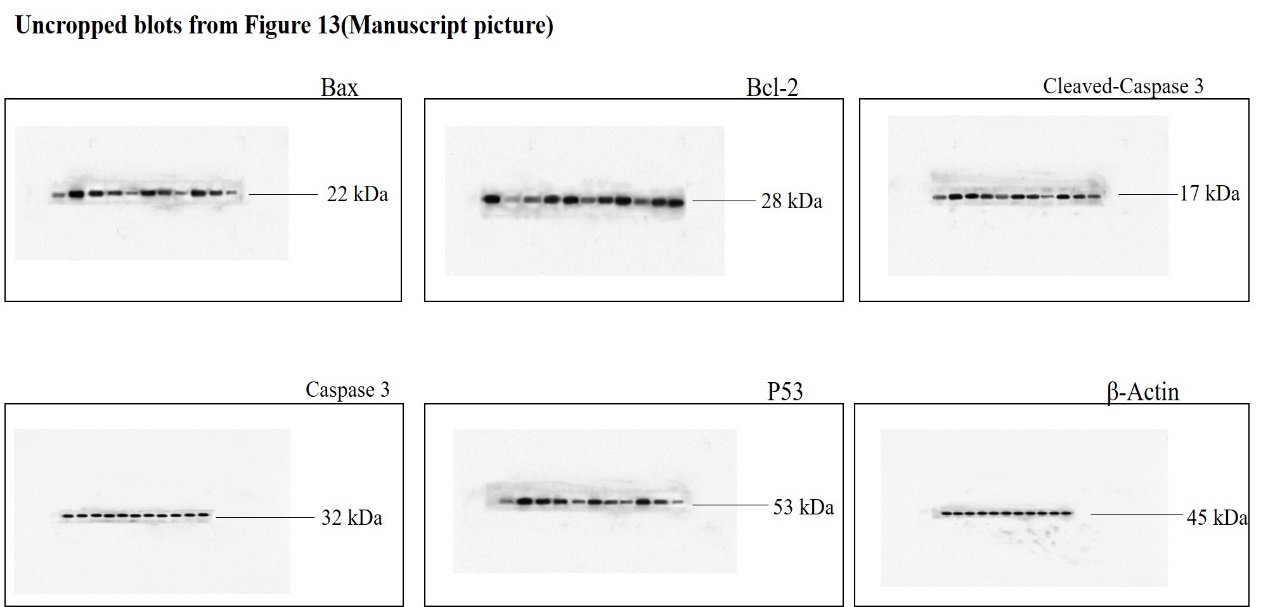


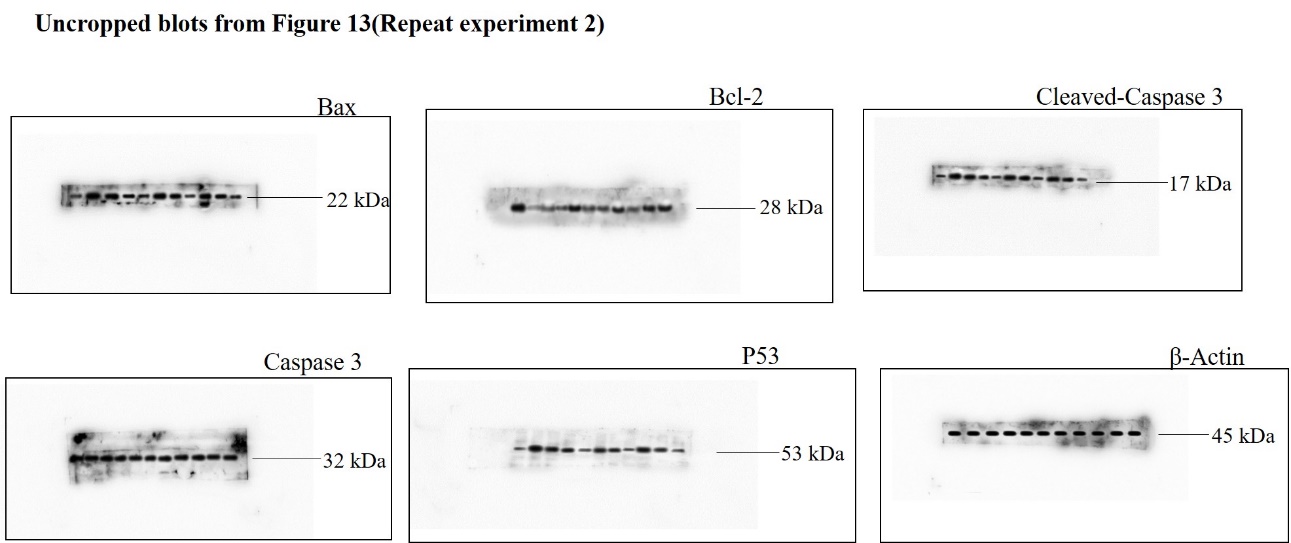


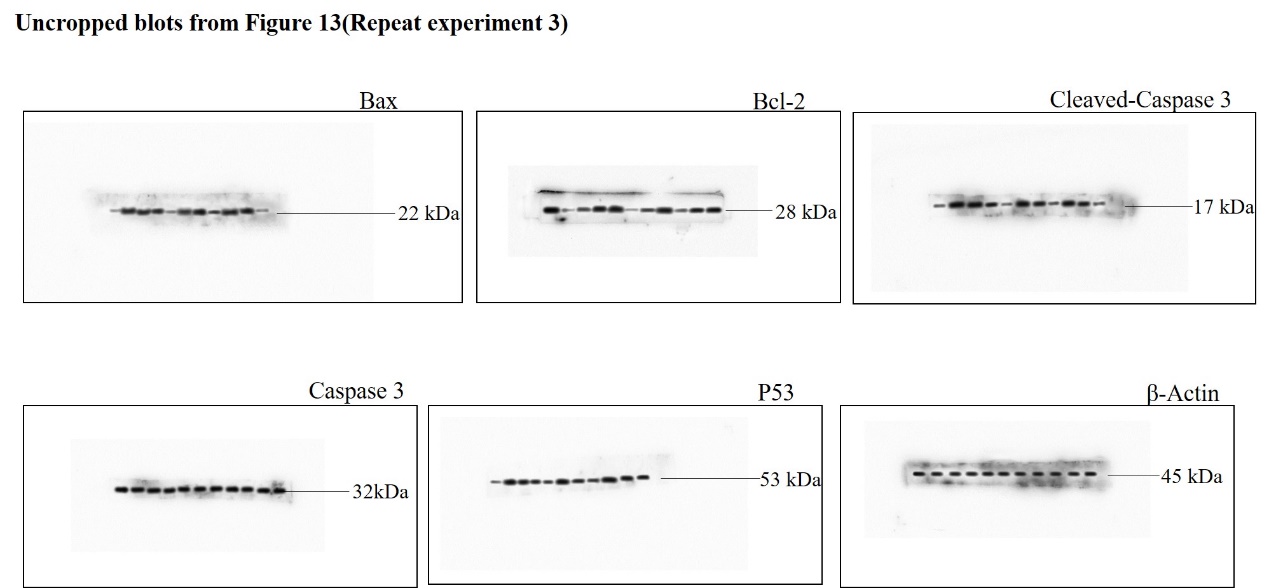


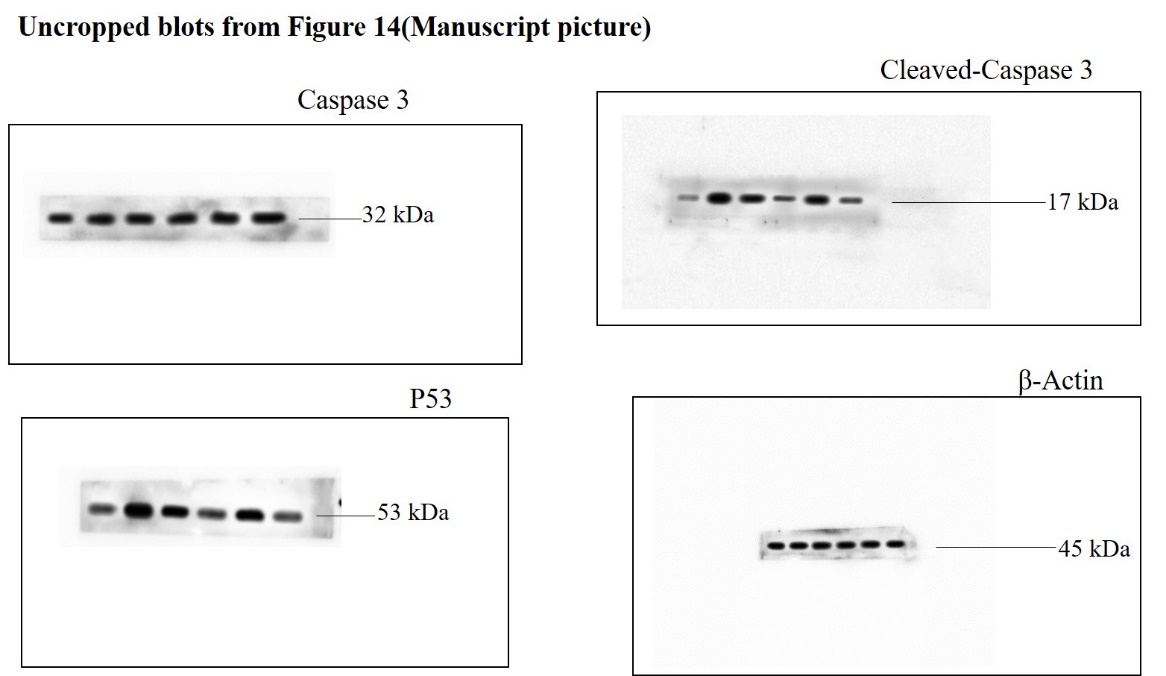


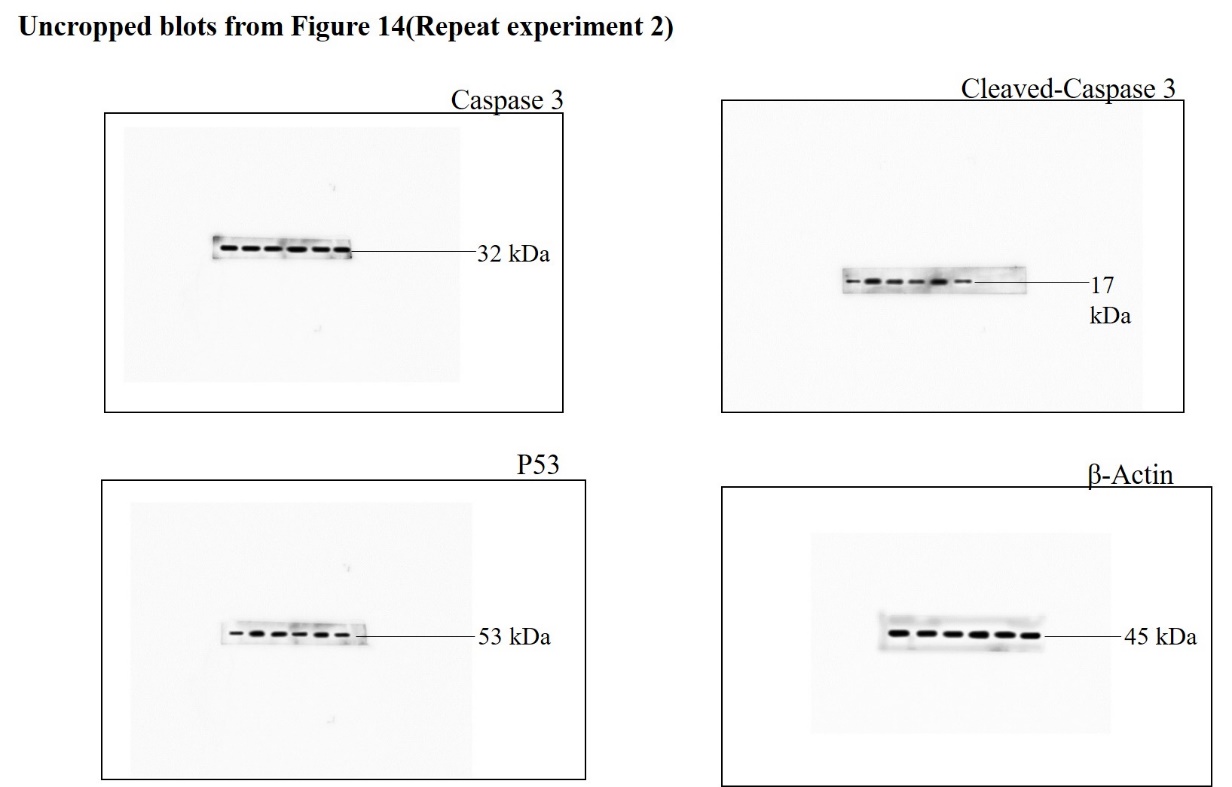


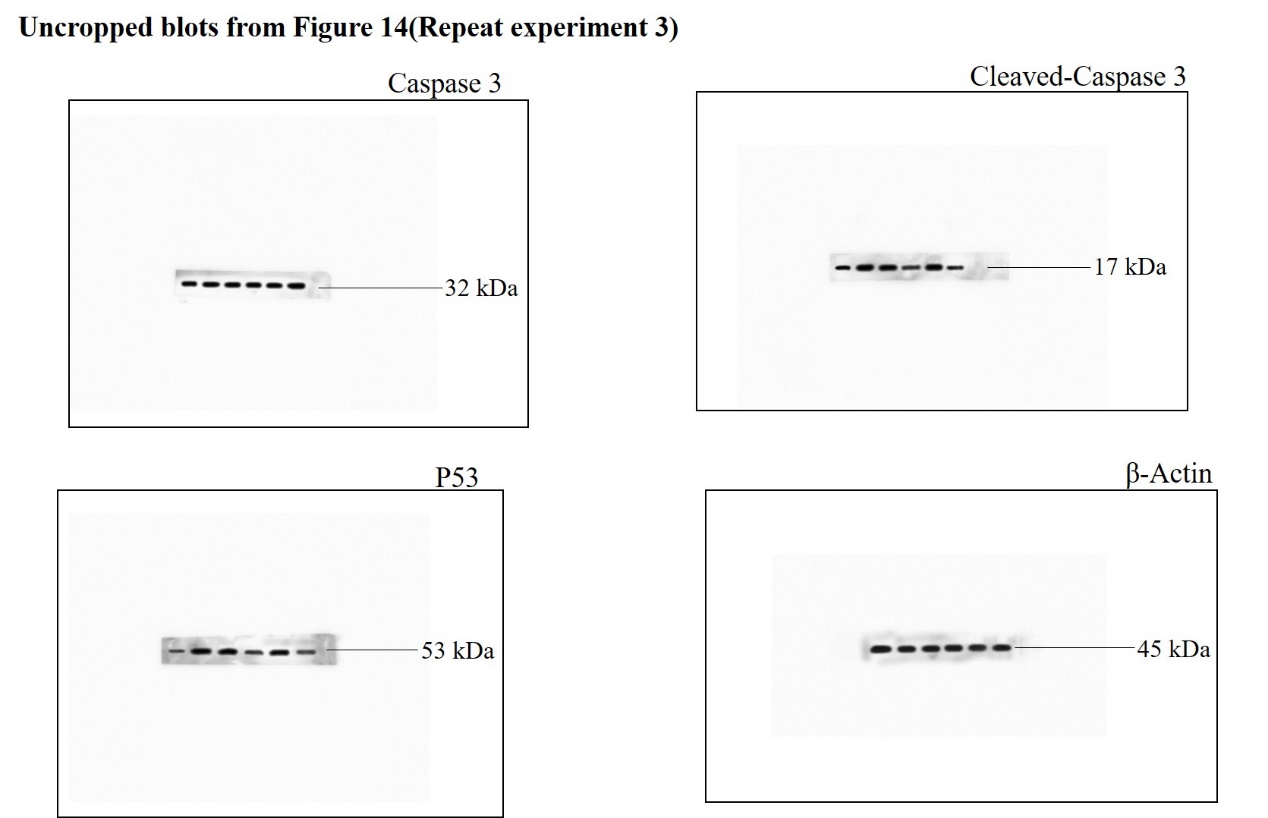


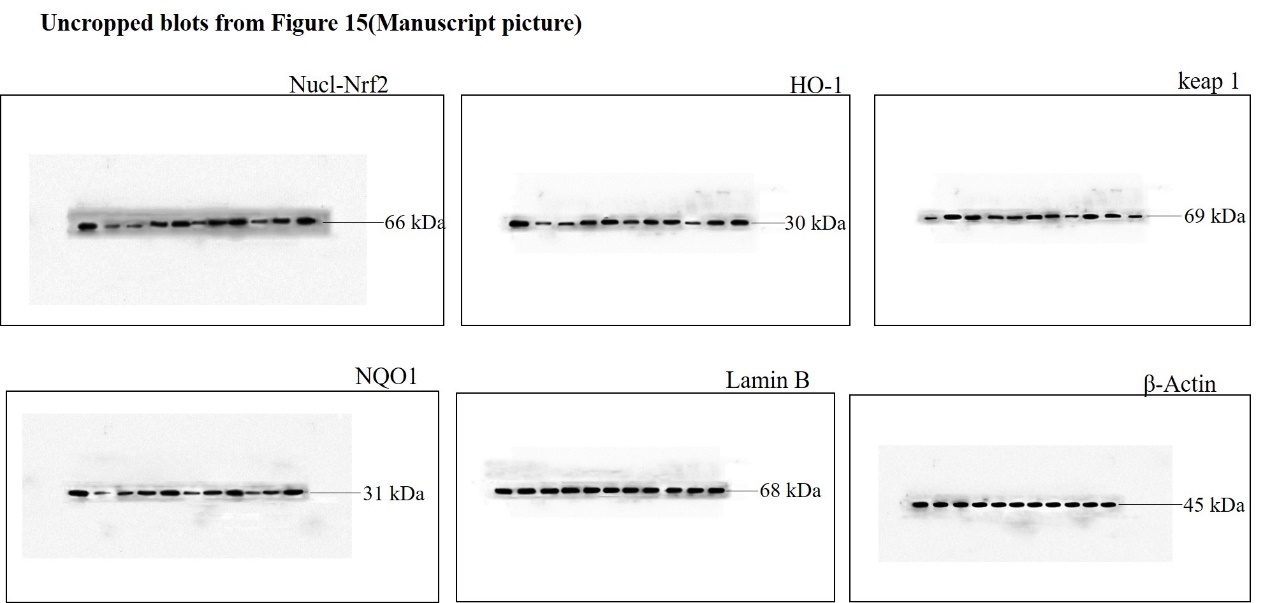


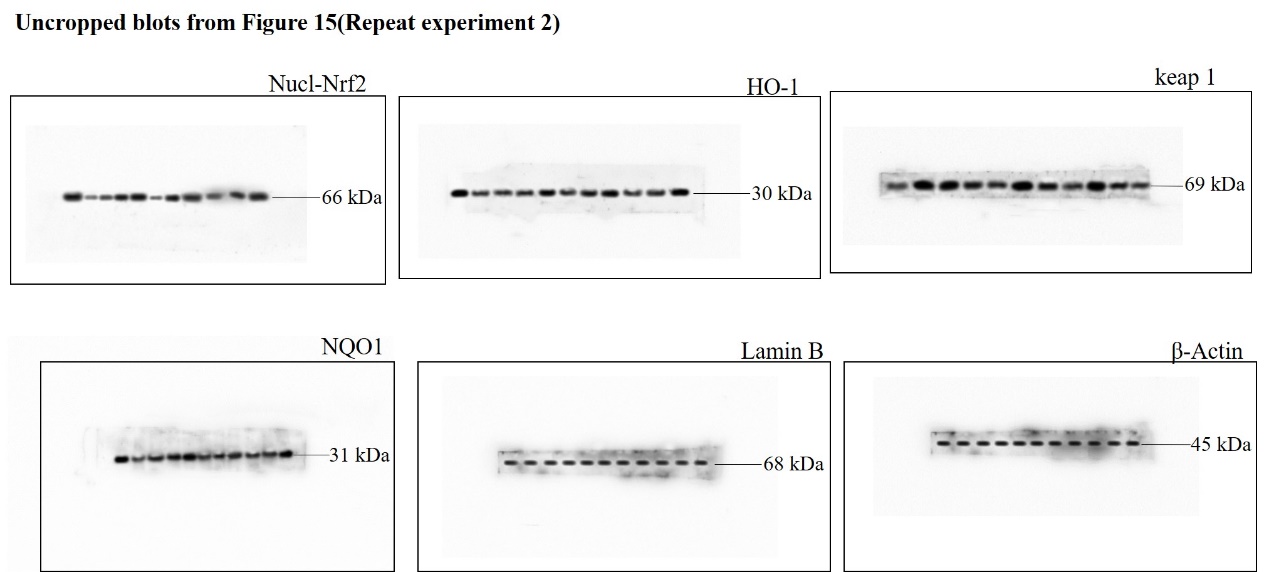


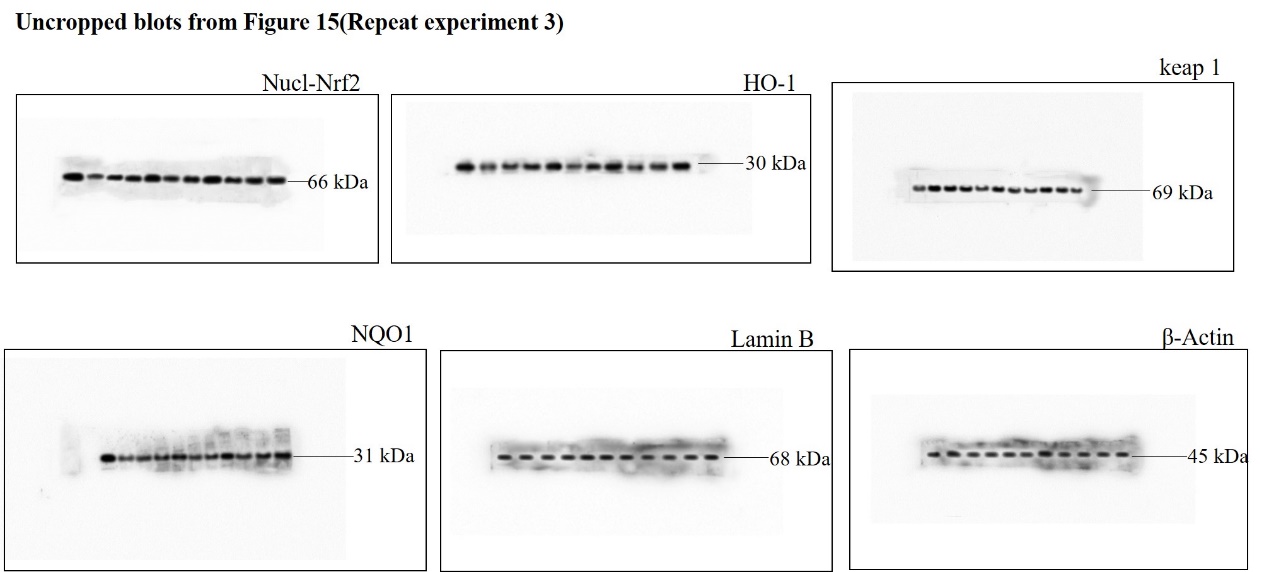


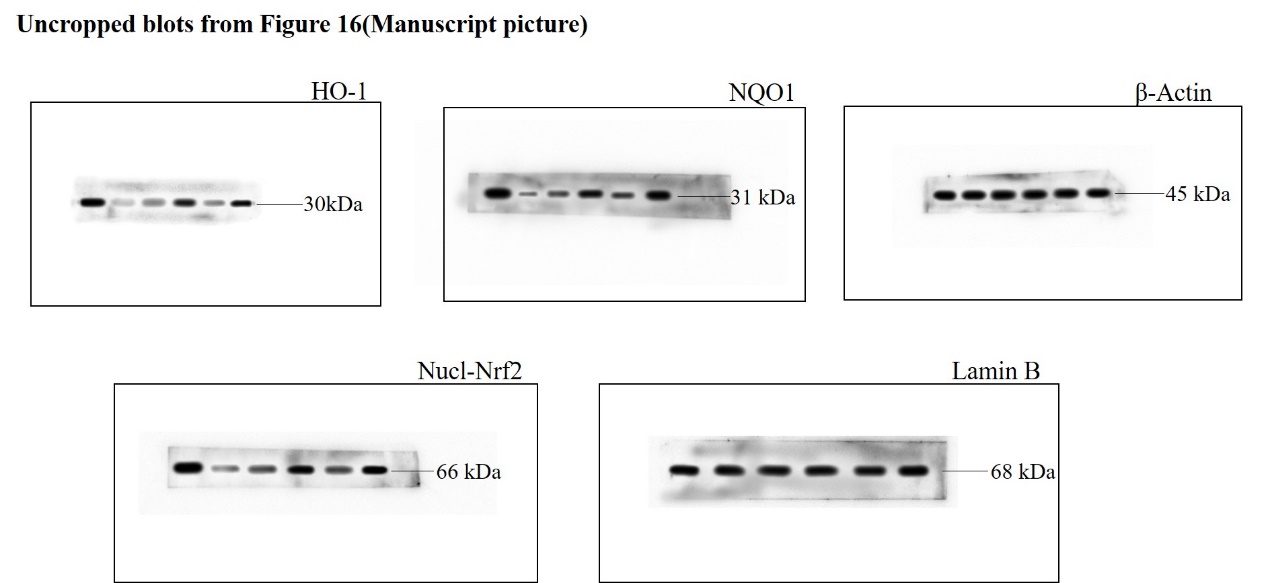


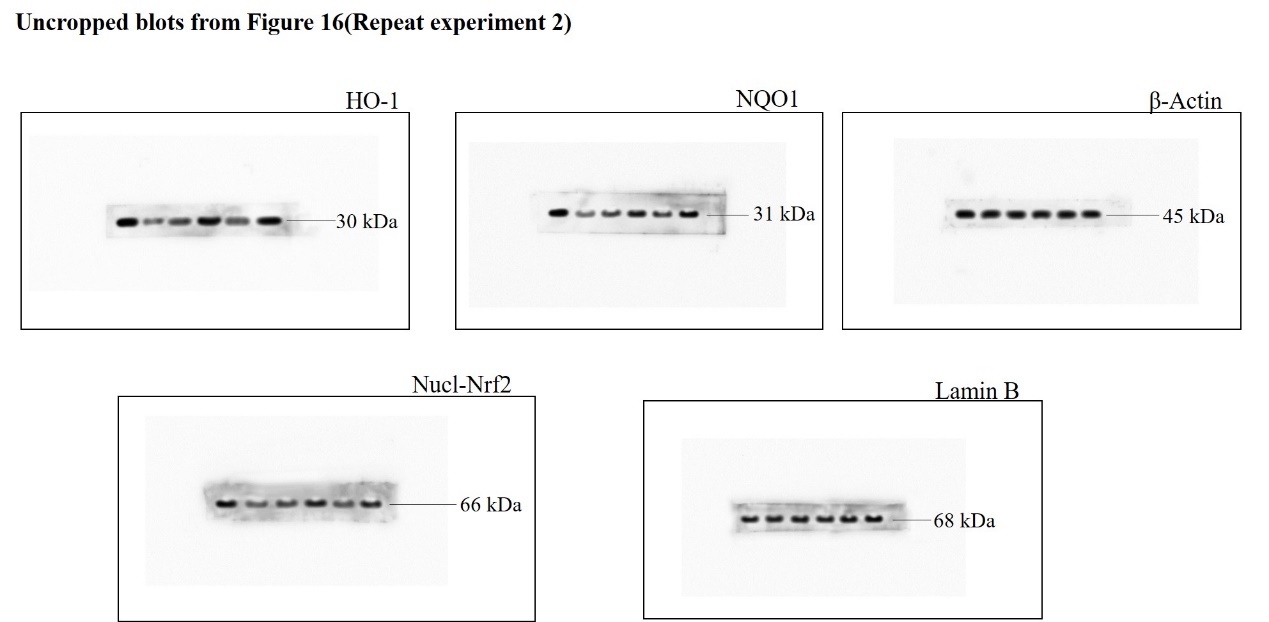


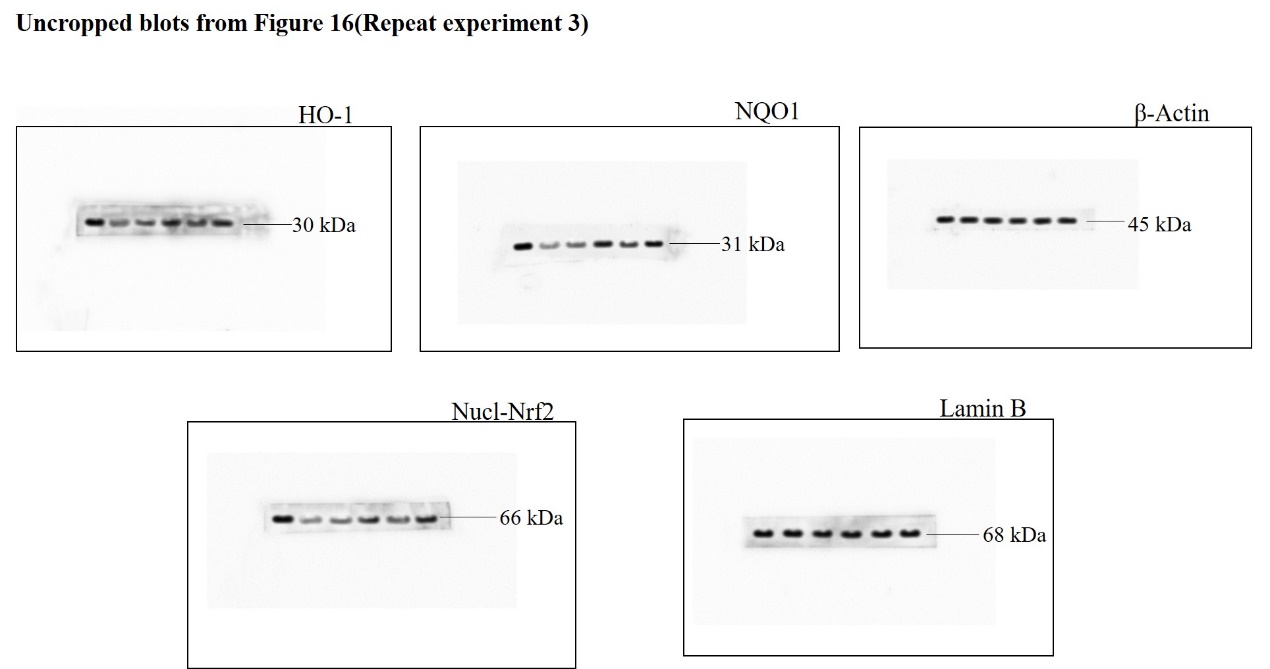


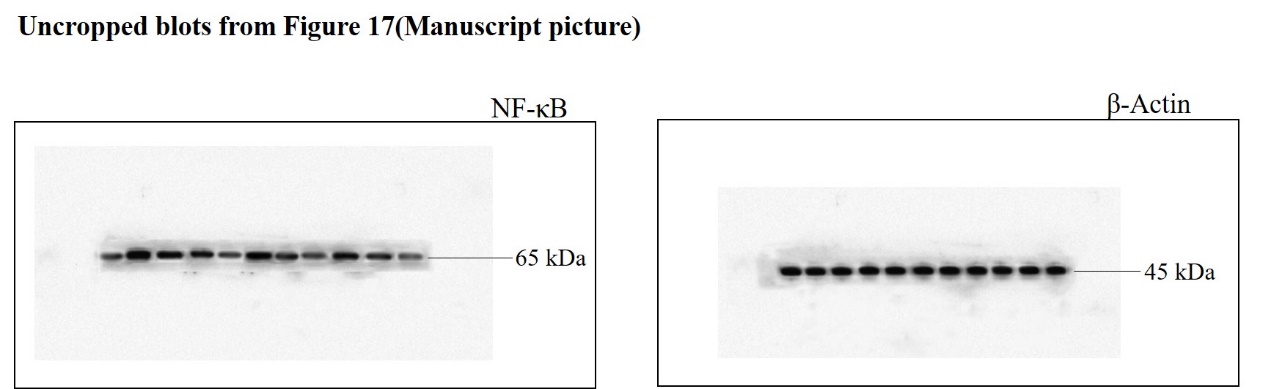


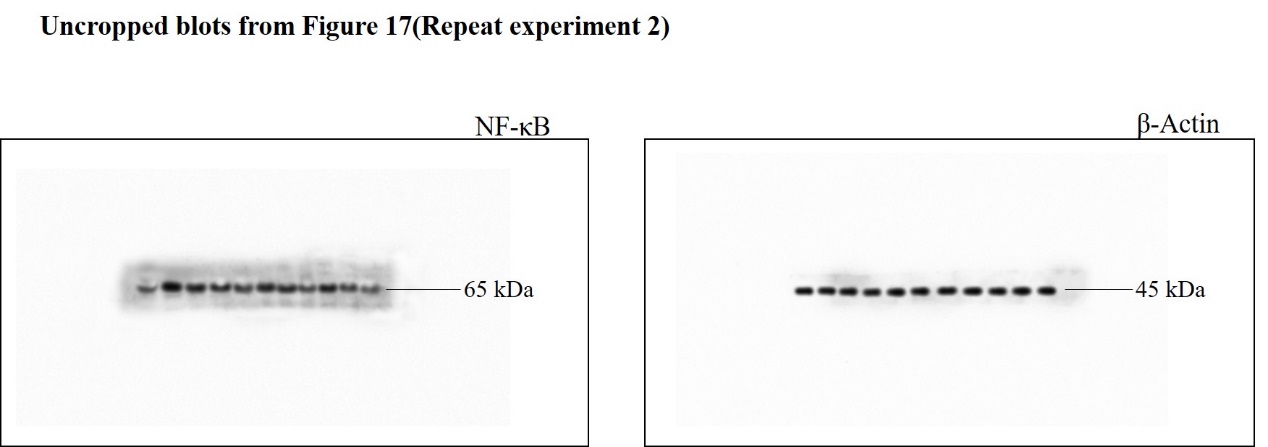


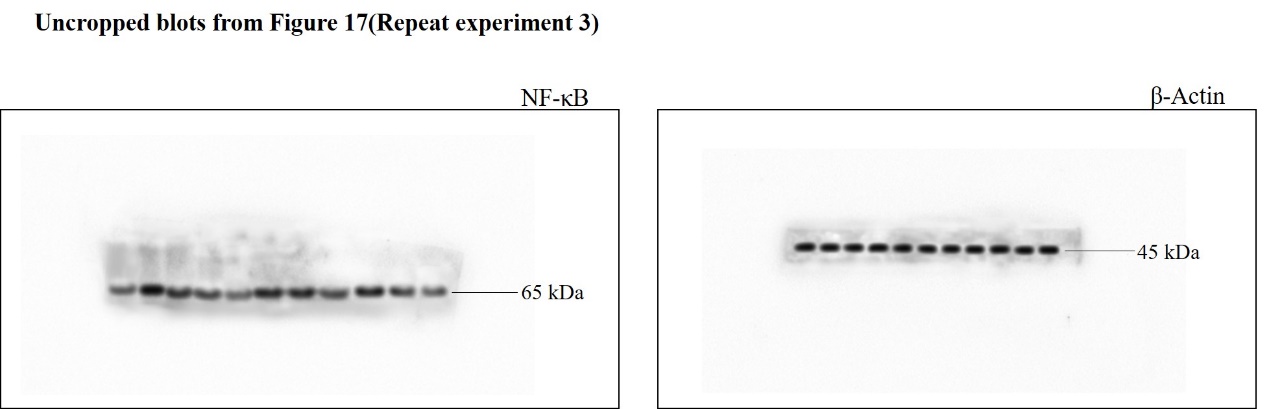


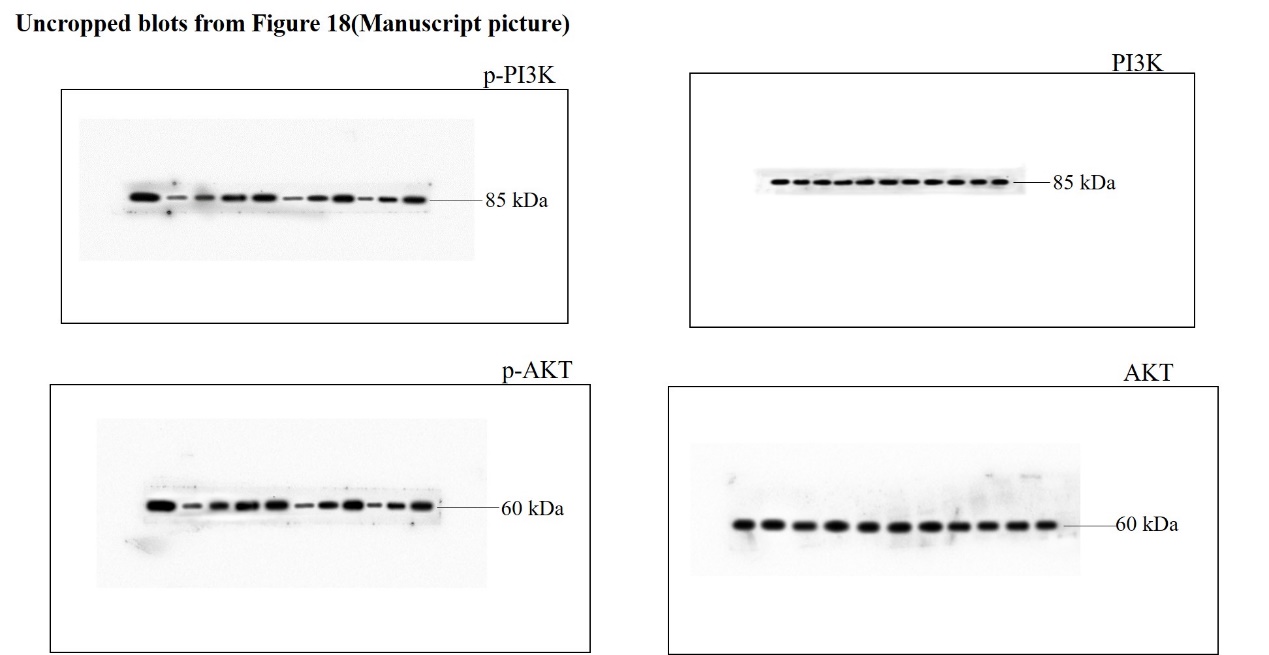


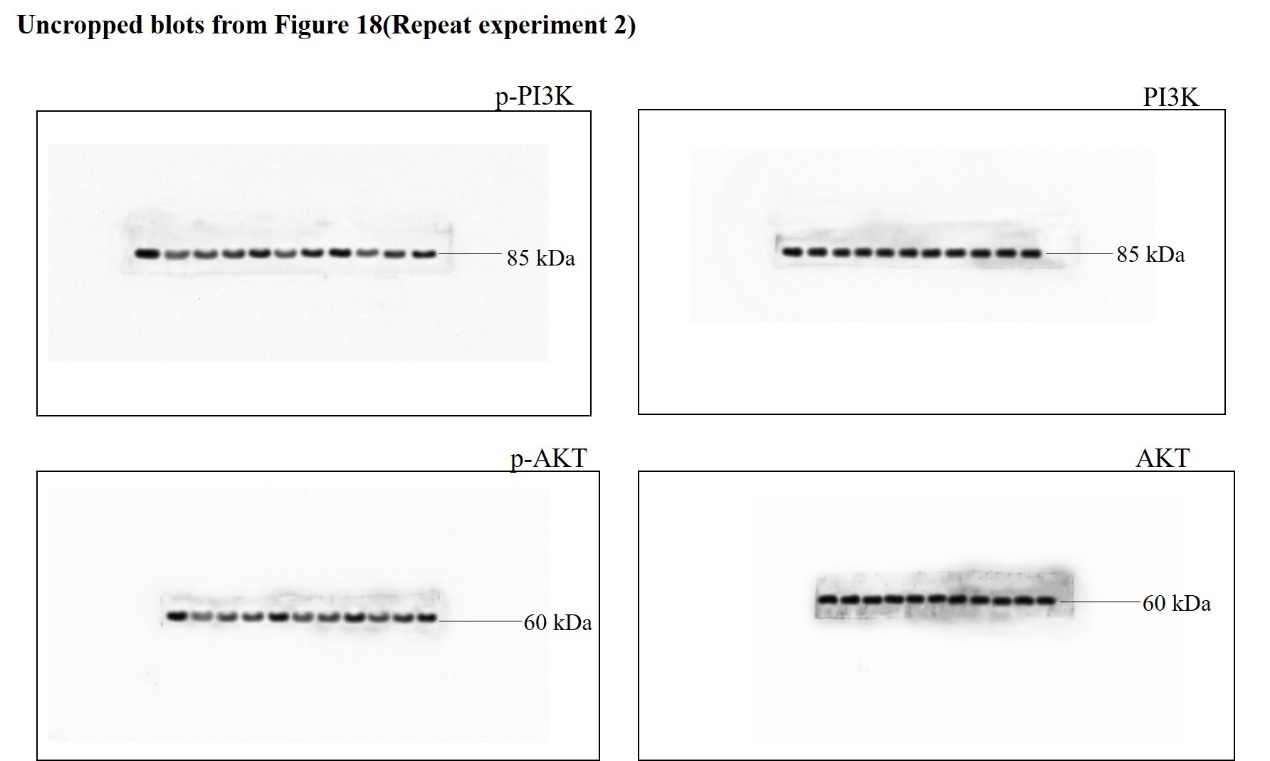


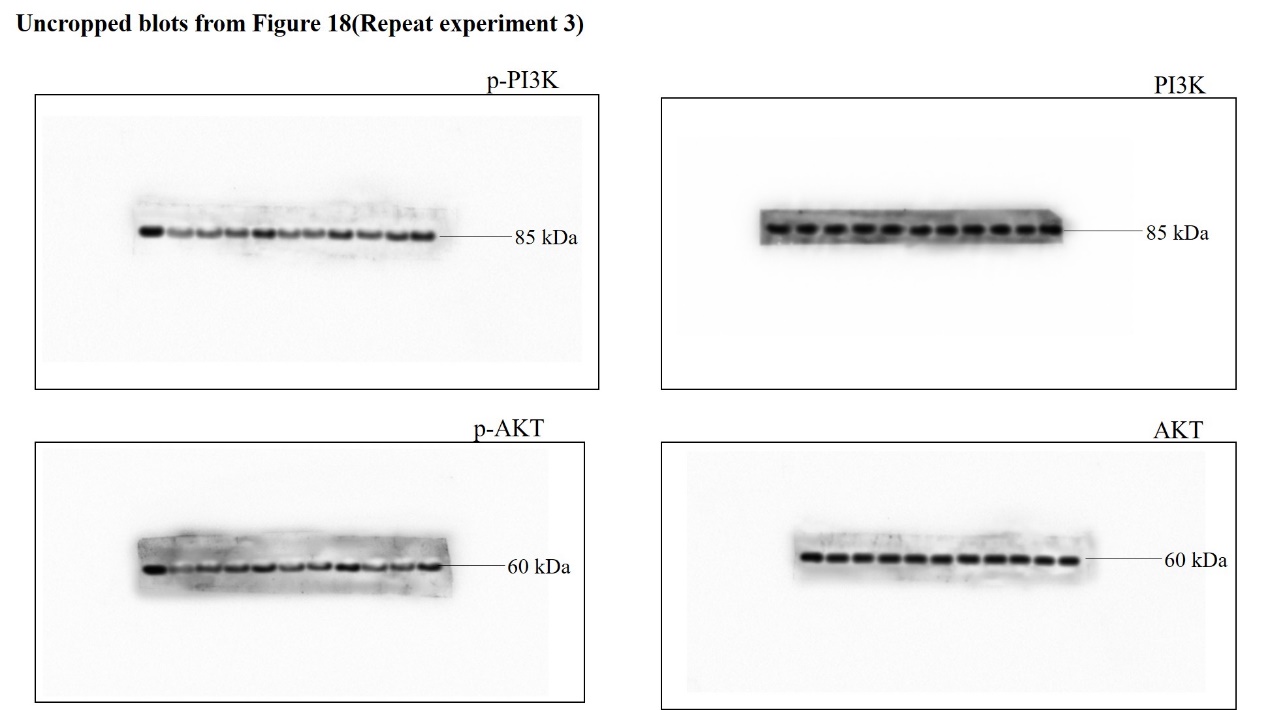

Supplement: Supplementary file 1 — Supplementary Information [file 41598_2020_71943_MOESM1_ESM.docx]
